# Novel Nontoxic 5,9-Disubstituted SN38 Derivatives: Characterization of Their Pharmacological Properties and Interactions with DNA Oligomers

**DOI:** 10.3390/ijms22158190

**Published:** 2021-07-30

**Authors:** Elżbieta Bednarek, Wojciech Bocian, Magdalena Urbanowicz, Jerzy Sitkowski, Beata Naumczuk, Lech Kozerski

**Affiliations:** 1 Falsified Medicines and Medical Devices Department, National Medicines Institute, 00-725 Warsaw, Poland; w.bocian@nil.gov.pl (W.B.); m.urbanowicz@nil.gov.pl (M.U.); j.sitkowski@nil.gov.pl (J.S.); b.naumczuk@nil.gov.pl (B.N.); l.kozerski@nil.gov.pl (L.K.); 2 Institute of Organic Chemistry, Polish Academy of Sciences, 01-224 Warsaw, Poland

**Keywords:** camptothecin, DNA complexes, molecular modeling, ^1^H/^13^C NMR, DOSY

## Abstract

Novel nontoxic derivatives of SN38 with favorable antineoplastic properties were characterized in water solution using NMR. The phenomena observed by NMR were linked to basic pharmacological properties, such as solubility, bioavailability, chemical and stereochemical stability, and binding to natural DNA oligomers through the terminal G-C base pair, which is commonly considered a biological target of Topo I inhibitors. Compound **1**, with bulky substituents at both C5(*R*) and C20(*S*) on the same side of a camptothecin core, manifests self-association, whereas diastereomers **2**, with bulky C5(*S*) and C20(*S*) substituents are mostly monomeric in solution. The stereogenic center at C5 is stable in water solution at pH 5–6. The compound with an (N-azetidinyl)methyl substituent at C9 can undergo the retro Mannich reaction after a prolonged time in water solution. Both diastereomers exhibit different abilities in terms of binding to DNA oligomers: compound **1** is strongly bound, whereas the binding of compound **2** is rather weak. Molecular modeling produced results consistent with NMR experiments. These complementary data allow linking of the observed phenomena in NMR experiments to basic preliminary information on the pharmacodynamic character of compounds and are essential for planning further development research.

## 1. Introduction

**Scheme 1:**
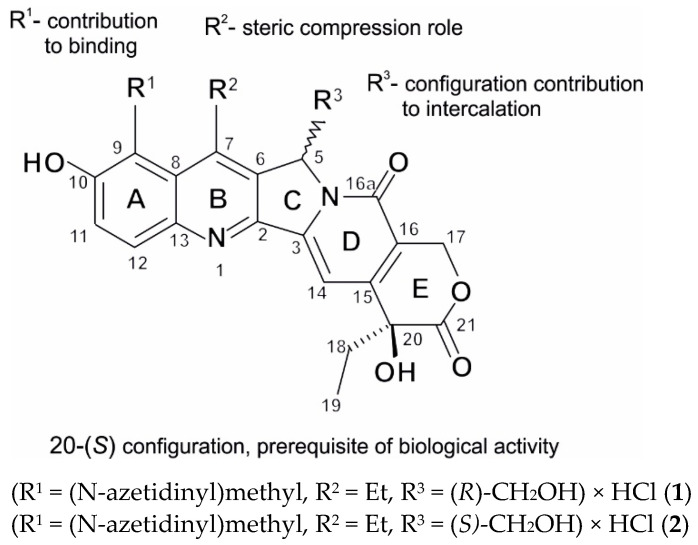
The structure of SN38 derivatives.

In biological studies, the DNA/topoisomerase I (Topo I) complex has been firmly established as a molecular target of anticancer drugs [1,2,3,4]. Potential Topo I inhibitors of the camptothecin family, currently in the clinical stage of development [5,6], are mainly the outcome of SAR studies [7]. The role of topoisomerase I inhibitors involves ternary complexes composed of enzyme/DNA/inhibitor [8]. Because studied compounds are considered for driving into further preclinical studies due to their favorable response to non-neoplastic cells, it was essential to compare their properties in a binary complex DNA/inhibitor with respect to their role as Topo I poisons in a ternary complex DNA/Topo I/inhibitor, by hindering the relegation of unstrained duplex of cancer DNA. The inhibitor interacts with both components of a ternary complex, the DNA, and an enzyme; therefore, the substituents around the camptothecin core (Scheme 1) contribute essentially to the final impact of an inhibitor on its biological activity. The cleavage site in DNA, formed via enzymatic action, is a molecular target for the inhibitor, and a substitution pattern in the camptothecin core is of primary importance. Earlier results have shown that depending on the nature and site of substitution in a camptothecin core, the substituent can play different roles in biomolecular interactions with nicked DNA. Certain SN38 derivatives with alkylamino substituents at position C9 can cause alkylation of DNA via formation of an o-methylene quinone intermediate with a neighboring hydroxy group [9,10,11]. The lack of an ethyl group at C7, as in topotecan, results in intercalation of 9-alkylamino-substituted camptothecins with natural [12] or nicked DNA oligomers [13]. The substituents on C5 can, in general, contribute to strengthening the intercalation.

With the aim of uncovering, in more detail, the mechanism of drug interaction with nicked DNA at a chemical molecular level, we synthesized several C5 and/or C9 substituted SN38 derivatives [14] bearing substituents that may have different roles in interactions with DNA oligomers. The absolute configuration of the substituent at the C5 carbon atom was assessed by electronic circular dichroism (ECD), and in vitro biological assays on several cancer cells and normal cells were performed.

The present contribution concerns one of the recently disclosed new camptothecin derivatives (Scheme 1), differing in absolute configuration at C5 and having promising properties allowing considering driving them into preclinical stage of development. The most important feature of a potential pharmaceutics is safety of a patient which is secured by the nontoxicity to normal cells. As the title compounds showed preferable IC50 of 0.33 and 0.11 µmol/L for **1** and **2**, respectively, for blood cancer cells, and appeared nontoxic against normal CRL 1790 cells [14], we decided to study their pharmacologic properties in more detail.

It was presumed that both obtained diastereomers, **1** (5*R*, 20*S*) and **2** (5*S*, 20*S*), with a cis or trans orientation of substituents at C5/C20 lying on the same or opposite side of a plane of the camptothecin core, respectively, may significantly differ in their interactions with the biological target. In the first instance, however, this steric interrelation between substituents may essentially influence the aggregation properties of both diastereomers in water, a phenomenon which was earlier observed and quantified for topotecan (TPT) [12].

In the present research, we conducted an NMR study of the interaction between novel diastereomeric derivatives of SN38 bearing C5(*R*)- or C5(*S*)- hydroxymethyl substituents, and the octamer duplex d(GCGATCGC)_2_ (**3**). There is a common consensus that active Topo I inhibitors stack the G-C base pair in a wild-type nicked DNA [15]. We therefore chose the model oligonucleotide with the ultimate G-C base pair for checking the mode of binding of both diastereomers, a feature which may influence their activity as potential Topo I inhibitors. The alkylamino substituent at C9 may, in principle, contribute to DNA alkylation, while the hydroxymethyl at C5 may influence intercalation, presumably by hydrogen bonding inside the nick. We deliberately chose d(GCGATCGC)_2_, which has the advantage of having G-C base pairs at both ends, mimicking one of the faces in nicked DNA [13,16]. It was earlier shown that the camptothecin core of TPT binds preferentially to this ultimate base pair [12].

In this paper, novel derivatives of SN38 were characterized in water solution. (5*R*, 20*S*) 1, was observed to easily aggregate even at millimolar concentration, whereas the other diastereomer, **2** (5*S*, 20*S*), is mostly monomeric in solution. The stereogenic center at C5 was shown to be basically stable in water solution at pH 5–6. The (N-azetidinyl)methyl substituent C9 underwent the retro Mannich reaction. Each diastereomer has a different strength of binding to d(GCGATCGC)_2_; compound **1** is strongly bound, whereas the binding of compound **2** is very weak. These data give basic preliminary information on the pharmacodynamic character of the compounds, which is essential for planning further research oriented at the development of pharmacologically useful compounds.

## 2. Results and Discussion

The compound 5(*R*)-hydroxymethyl-7-ethyl-9-(N-azetidinyl)methyl-10-hydroxy- camptothecin and its diastereomer 5(*S*) were obtained as formate salts using one-pot synthesis (see also the Supplementary Materials—SM) [14]. Their RT values as determined by HPLC were 5.7 and 11.0 min for 5(*R*) and 5(*S*), respectively (see the HPLC of the reaction mixture in Figure S1, in the SM). Then, the compounds were separated and purified by HPLC and converted into hydrochlorides **1** ((5*R*, 20*S*)) and **2** ((5*S*, 20*S*)).

**Figure 1:**
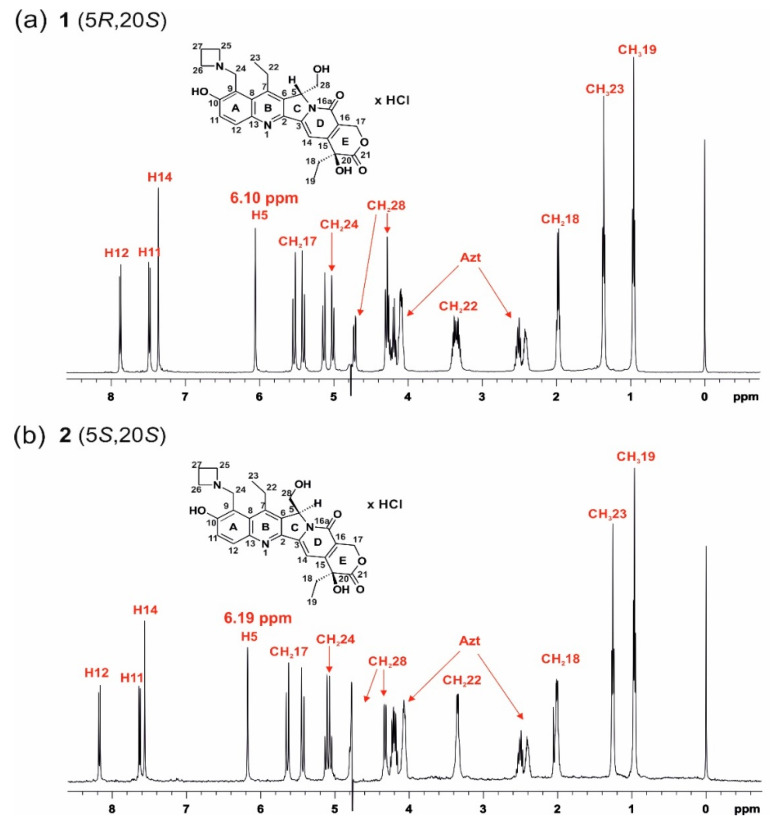
The ^1^H NMR spectra of diastereomers: (**a**) **1** and (**b**) **2** in D_2_O solution, pH 5, temp. 25 °C.

**Figure 2:**
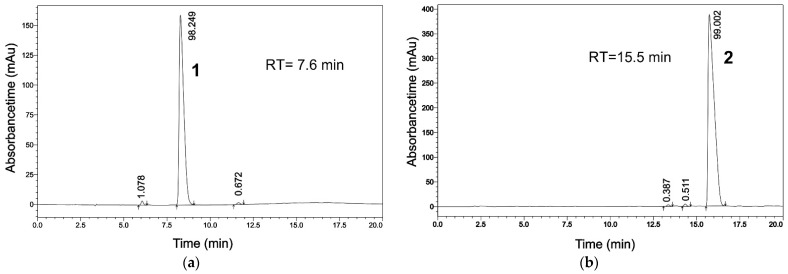
HPLC chromatograms of diastereomers: (**a**) **1** and (**b**) **2** (for HPLC conditions, refer to the SM).

The ^1^H NMR spectra of both diastereomers, **1** and **2**, are presented in Figure 1. They differ distinctly, particularly in the aromatic and azetidine region of the signals (see Tables S1 and S2 in the SM). The configuration at C5 may be easily distinguished by the chemical shift difference of the C5-H proton, i.e., 6.10 and 6.19 ppm for C5(*R*)-H and C5(*S*)-H, respectively (Figure 1), and by the RT in HPLC. Figure 2 shows HPLC chromatograms of compounds **1** (RT: 7.6 min) and **2** (RT: 15.5 min) after purification.

### 2.1. Chemical and Stereochemical Stability of ***1*** and ***2*** in Water

**Figure 3:**
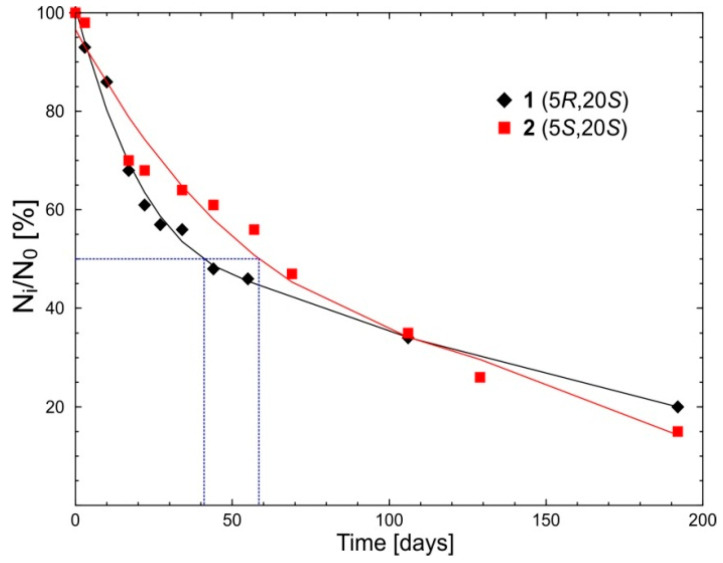
Kinetic graph showing the degree of degradation (Ni/N_0_) of diastereomers **1** and **2** in D_2_O, pH 5, temp. 25 °C, over time. To determine the degree of degradation (Ni/N_0_), the integrals of chosen Scheme 1. H NMR spectra were monitored during a given period. Ni is the average integral of selected signals in the ^1^H NMR spectrum, recorded at the i-th experimental point, and N_0_ is the average integral of selected signals in the ^1^H NMR spectrum recorded at the beginning of the experiment.

The property of chemical and stereochemical stability in water is crucial for a potential medicine that needs to reach its biological target in an unchanged form. The results of kinetic experiments, which reflect the stability of both diastereomers at pH 5 in water solution, are shown in Figure 3. This allowed estimation of the half-life time t½, which is similar for both diastereomers.

After ca. 50 days, 50% of both diastereomers were transformed. The ^1^H and ^1^H–^13^C HSQC and ^1^H–^13^C HMBC NMR spectra were obtained to identify the products of transformation (see Figure S2 and Tables S1 and S2 in the SM). It should be noted that direct carbon spectra are not available due to the low solubility of the compounds under investigation. The most important data are marked in red in both tables, as they concern the substituents at the C5 and C9 carbon atoms. In the case of the product of the transformation of **1**, for the C9 carbon atom at 108.58 ppm, only a weak correlation with proton H11 was found in the HMBC spectrum. In addition, the peak at 108.58 ppm was not observed in the HSQC spectrum. Taking into account the above observations, we can suggest that substitution of the C9 carbon atom with deuterium, as a result of the retro Mannich reaction of compound **1** in D_2_O, has occurred. Due to the low solubility and, therefore, sensitivity reasons, the product of the transformation of diastereomer **2** was not confirmed by the HMBC spectrum. Therefore, the incubation of **2** was repeated in H_2_O phosphate buffer (25 mM NaCl/25 mM K_3_PO_4_, pH 6). Under these conditions, the transformation was nearly complete within a few days and allowed for the full assignment of ^1^H and ^13^C spectra (see Table S3). The appearance of a proton signal attached to the C9 carbon atom (^1^H–^13^C HSQC spectrum) confirmed the same process as in **1**. Thus, the main products of the retro Mannich transformation of **1** and **2**, under the assumption of unchanged chirality, are 5-(*R*)-hydroxymethyl-7-ethyl-10-hydroxycamptothecin (**1A**) and its diastereomer 5-(*S*) (**2A**), respectively.

**Figure 4:**
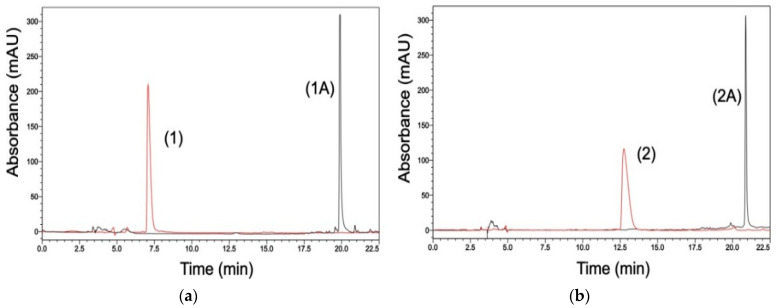
HPLC analysis of incubation **1** (**a**) and **2** (**b**) in D_2_O buffer (25 mM NaCl/25 mM K_3_PO_4_), pH 6, temp. 25 °C. In each panel, the red run demonstrates the starting point (**1** or **2**) and the black run the end of incubation, showing the products assigned as 5-(*R*)-hydroxymethyl-7-ethyl- 10-hydroxycamptothecin **1A** or its diastereomer 5-(*S*) **2A**. The fraction retention times were 7.4 min (**1**), 12.5 min (**2**), 19.8 min (**1A**), and 21.0 min (**2A**).

The confirmation of stability of the C5 chiral center requires more attention, because the ^1^H and ^13^C chemical shifts of both diastereomers are very close and depend on the concentration (see Section 2.2: Aggregation Studies of **1** and **2** in D_2_O Buffer). Therefore, the chirality of **1**, **2**, **1A**, and **2A** was independently confirmed by HPLC analysis. The HPLC chromatogram of the reaction mixture which contains both diastereomers **1** and **2** and the two SN38 derivatives monosubstituted at C5 with hydroxymethyl group in opposite absolute configurations **1A** and **2A**, established by ECD (vide infra), is presented in Figure S1 (see the SM). It can be seen that the RT values differ distinctly for the two pairs of compounds. Figure 4 shows the HPLC results before and after incubation of each diastereomer, **1** and **2**. The comparison of the RT values in Figure S1 and Figure 4 indicates that the products of transformation, **1A** and **2A**, have the same configuration at C5 as the starting diastereomers **1** and **2**, respectively. Moreover, it was concluded that the studied SN38 derivatives 1 and 2 retained a stable absolute configuration at the C5 carbon atom during the long period of incubation in water solution.

### 2.2. Aggregation Studies of ***1*** and ***2*** in D_2_O Buffer

In order to assess the influence of the geometry of both diastereomers on their association abilities in water solution, which can be linked to their bioavailability, we performed dilution experiments by ^1^H NMR and DOSY studies.

**Figure 5:**
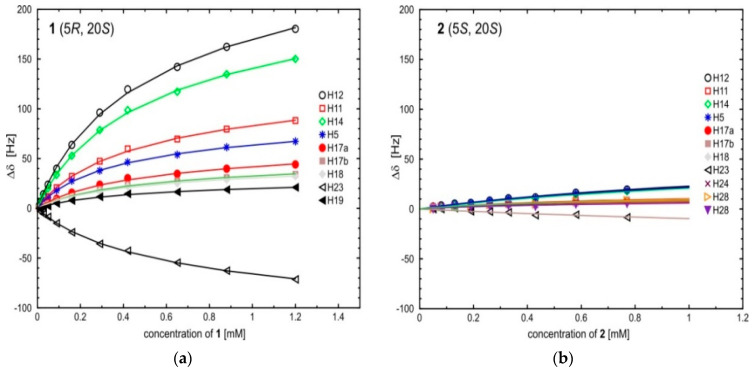
The chemical shift changes Δδ [Hz] of the proton signals of **1** (**a**) and **2** (**b**) as a function of concentration [mM] (D_2_O buffer, 25 mM NaCl/25 mM K_3_PO_4_, TSPA-*d_4_*, pH 6.4, temp. 10 °C). Positive values correspond to a low frequency shift.

The ^1^H NMR data for the dilution experiments are presented in Table 1 (for more details, see Tables S4 and S5 in the SM) and plotted in Figure 5. For both compounds, all protons, except H23, were shifted to lower frequencies. This result confirms our expectation that the mode of self-association of the studied compounds is stacking of the camptothecin core of monomers. The most noticeable differences are the shifts of aromatic ring protons in diastereomer **1**, compared with much smaller shifts of aliphatic protons on ring E (see Table 1 and Table S4 in the SM). This effect can justify the tentative suggestion that molecules of **1** self-associate face to face via rings A. This stands as the suggested rationale for the fact that both bulky substituents in **1**, i.e., (*R*)C5-CH_2_OH and (*S*)C20-C_2_H_5_, are on the same side of the camptothecin core, making the close stacking of opposite sides possible. A special note requires the large changes in the chemical shifts of protons H12 and H14 during self-association (calculated maximum Δδ for the association process exceeding 300 Hz (see Table 1). This suggests strong stacking interactions in a uniform mode, face to face, with rings A. The reverse shift of the methyl group 23-CH3 can also be rationalized within this model because it is forced to accommodate this along with the camptothecin core, in the deshielding of the aromatic rings. The observed chemical shift changes were much smaller for **2** (see Table 1 and Table S5 in SM). This is not unexpected given the above, because both bulky groups are situated on opposite sides of the camptothecin core, thus making the stacking of monomers less favorable.

The self-association of both diastereomers was confirmed in diffusion experiments, performed on samples with selected concentrations (Table 2). It was observed that in both cases, the value of the Di coefficient increased at a lower concentration, reflecting the dissociation process of self-aggregated species.

The average values of the self-association constants K_a_, calculated based on dilution experimental data, were 1.71 ± 0.24 and 0.70 ± 0.38 mM^−1^ for **1** and **2**, respectively (Table 1). These values can be compared with the much higher self-association constant K_a_ 3.4 ± 1.0 mM^−1^ for topotecan in a similar solution, at 30 °C [12]. This can be attributed to the lack of C5-CH_2_OH and C7-C_2_H_5_ groups in the case of TPT, meaning steric hindrance is not introduced to the stacking, explaining the differences in self-association of compounds in the present study. The observed chemical shift changes (Figure 5), as well as the calculated association constants, indicate that **1** is more strongly associated than **2**.

### 2.3. Binding of ***1*** or ***2*** with d(GCGATCGC)_2_, Characterized by DOSY

Due to the different geometry of diastereomers **1** and **2**, one could expect different affinities to the model DNA d(GCGATCGC)_2_. Therefore, DOSY experiments were performed at different molar ratios of **1** or **2** to d(GCGATCGC)_2_. The data are given in Table 3.

To determine the diffusion coefficient from the PFGSE spectra of **1** or **2** in the presence of octamer **3** only, no overlapped signals were used; therefore, we could secure the calculation of the true binding constant in the PFGSE experiment.

Here, we assumed that the mode of interaction involves a monomer of **1** or **2** interacting with the ultimate G1-C8 base pair of d(GCGATCGC)_2_ (vide infra). This is justified if we recall the established self-association constants of both diastereomers being much weaker (1.7 and 0.7 mM^−1^ for **1** and **2** ,respectively, vide infra) than the presently discussed binding constants with octamer **3**. The calculations presented in Table 3 show that, regardless of the ratio of interacting compounds, in each case the affinity constants are different for both diastereomers. The comparison of items **1** and **2** in Table 3, marked in red, is relevant because in both solutions there is a similar 1:3 ratio of interacting species. The binding constant for **1** is three times larger, and the mole fraction of the complex is also much higher. Interestingly, the binding constant of topotecan to the same octamer is only 1.7 mM^−1^ [12], close to the value obtained for diastereomer **2**.

### 2.4. Mode of Binding of Diastereomers ***1*** and ***2*** to d(GCGATCGC)_2_ Based on ^1^H NMR Titration

**Figure 6:**
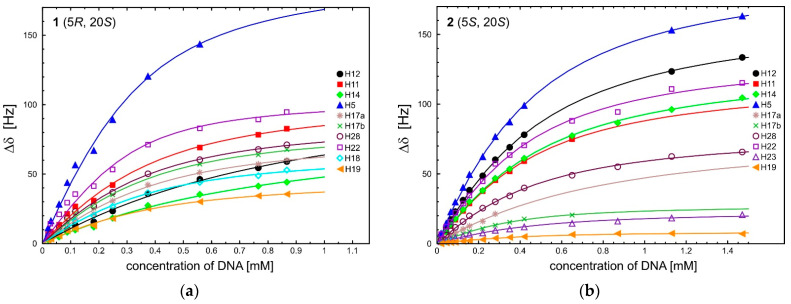
The chemical shift changes of the proton signals of **1** (**a**) and **2** (**b**), Δδ [Hz], during titration by octamer **3 (DNA)**, as a function of the concentration of **3** [mM]; D_2_O buffer, (25 mM NaCl/25 mM K_3_PO_4_), pH 6.0, temp. 10 °C.

The diffusion experiment, DOSY, allowed us to establish the strength of binding, but not the mode of binding, which can be derived from the quantification of chemical shift changes induced on proton signals of a camptothecin core upon titration with increasing amounts of **3**. The numerical data are listed in Table 4 (for more detail, see Tables S6 and S7 in the SM) and plotted in Figure 6. The induced chemical shift changes for some protons of d(GCGATCGC)_2_ interacting with the aromatic core of **1** and **2** are cited in Table 5. It can be seen that the largest shifts were induced on protons in an ultimate G1-C8 base pair by the camptothecin core, which suggests that both interacting molecules are stacking because all signals, except one, were shifted to lower frequencies. The shifts induced by diastereomer **1** were much larger than by diastereomer **2**. This could be expected given the established earlier fact (Table 3) that diastereomer **1** has a higher population of molecular complexes.

In the absence of cross-peaks between octamer **3** and **1** or **2** in the NOESY spectra, preventing discussion of the intermolecular proton distances, the mode of stacking can be judged from the titration shifts induced on a camptothecin core by titrating the diastereomers with an increasing amount of d(GCGATCGC)_2_ (Table 4). While the NOESY cross-peaks depend mainly on the interproton distances, allowing their direct connection within the molecular geometry, the chemical shift of a given proton in solution is dependent on several factors: in the present case, self-association, magnetic anisotropy effects of aromatic rings or carbonyl groups, and hydrogen bonding. In the present study, the major factor influencing the chemical shift changes is the mutual shielding effect of the aromatic rings of both stacking molecules. The data showing low frequency shifts of camptothecin protons, except for H19 of **1**, induced by stacking of the G1-C8 base pair, are given in Table 4 and plotted in Figure 5. The analytical equation describing the binding isotherm for each proton, formulated in terms of the chemical shift, is given in the Experiment section. Table 4 presents the binding constants of both diastereomers. Their values are in agreement, within the standard error, with those calculated from the diffusion experiment. The values marked in red for diastereomer **1** require a comment on the method of calculation. The basic parameter used in the calculation of K_a_ values is Δδ, which contains δ**_A_**, the chemical shift of a neat ligand monomer at a given concentration. Unlike the majority of protons in diastereomer **1**, for which chemical shifts are influenced mainly by stacking, the chemical shifts of protons H12 and H14 depend very strongly on self-association (comp. Figure 5). With the increasing concentration of DNA oligomers attracting the monomer of a ligand and causing its dissociation, the value of δ**_A_** in solution changes and, thus, drastically influences the shape of an isotherm for these protons at low and higher concentrations of octamer **3**, leading to the observed differences in the K_a_ value.

The data in Table 4 are also compatible with the structures from molecular modeling. It can be seen in Table 4 that the maximum chemical shift changes Δδ_max_ induced by stacking are large for the protons in rings A, B, and C of camptothecin (data marked in blue), but much smaller for ring E, which falls off the shielding cone of aromatic bases. This provides additional confirmation of the proposed mode of interaction, being similar for both diastereomers in the most abundant conformational clusters.

### 2.5. Molecular Modeling

**Figure 7:**
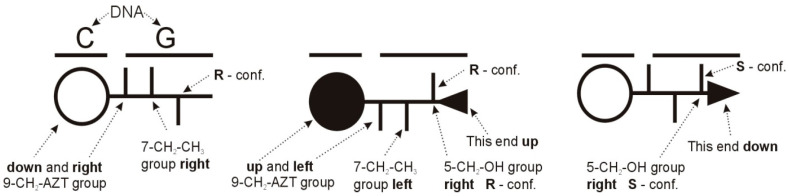
Schematic representation of the conformational geometries of diastereomers **1** and **2** vs. the ultimate base pair G1-C8.

**Figure 8:**
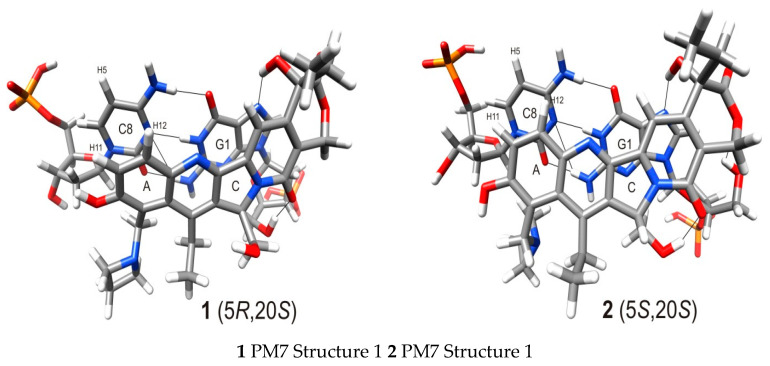
Molecular model of MD of the most abundant structures of the complexes of the ultimate base pair G1-C8 of a self-complementary duplex d(GCGATCGC)_2_ with diastereomers **1** (see Table 6, No. 1) and **2** (see Table 7, No. 1). For both structures, the hydrogen bonds of the base pairs and intermolecular C20-OH to N7 of the imidazole ring are shown. The additional intermolecular hydrogen bonds in the structure R-1 are shown between the CH_2_OH of G1 2′-deoxyribose and C16a carbonyl.

**Figure 9:**
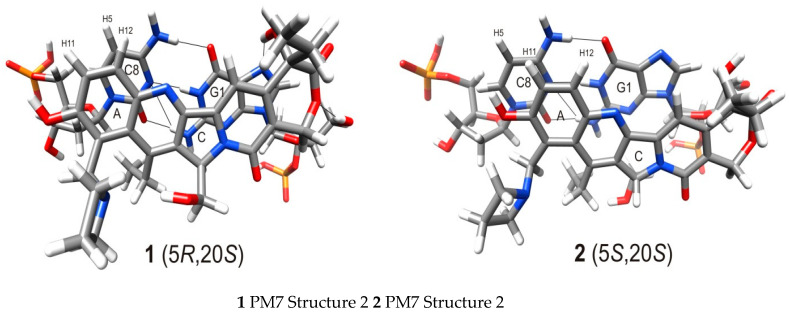
Molecular model of MD of the less abundant structures of the complexes of the ultimate base pair G1-C8 of a self-complementary duplex d(GCGATCGC)_2_ with diastereomers **1** (see Table 6, No. 2) and **2** (see Table 7, No. 2). Base-paired hydrogen bonds are shown. Both substituents on C5 and C20 point outward from the molecular complex in **1**, but the C5 substituents in **2** point towards the molecular complex space.

The studied compounds have several degrees of conformational freedom; 9-(N-azetidinyl)methyl, 7-ethyl, 5-(*R* or *S*)-hydroxymethyl and 20-*S*-ethyl and, therefore, a number of conformations were found using advanced molecular dynamics or the semi-empirical approach PM7 (see the SM, Tables S8–S11). Figure 7 shows the schematic presentation of the overall molecular shape of the molecules, which is clearly transposed on the molecular structures shown in Figure 8 and Figure 9. Nevertheless, here, we focus our discussion on the most abundant families of cluster conformations for **1** and **2**. These are shown in Table 6 and Table 7. In the case of diastereomer **1** (see SM Table S8), three of the most abundant families (51.8%) have the same parallel alignment with the G1-C8 base pair, and differ slightly in energy. The same parallel alignment has a family of three conformations (items 4, 7, 8 in Table S8, 13.5%), with ring E of diastereomer **1** tilted upward over the face of the G-C base pair (center pictogram in Figure 7). In the case of diastereomer **2** there is more diversity in the conformational families; two of the most populated (36.5%), similar to those in diastereomer **1**, are shown in Table 6. The second most populated family (22.6%, items 3, 5, 7, 9 in Table S9) has parallel interacting molecules, but the camptothecin core is tilted 180° such that ring E faces the C8 base and is tilted upward in three cases (items 3, 7, 9).

Here, we discuss only the first two clusters for both diastereomers in Figure 8 and Figure 9. It can be concluded that within that ensemble of clusters, there is more diversity of conformations in diastereomer **2**, which is less strongly bound and has more steric requirements for stacking with the G-C base pair than diastereomer **1**. This was confirmed by the binding isotherms in Figure 6, which are more spread for protons in **2** than in **1**, suggesting the latter has more definite conformations in the most populated clusters. Nevertheless, the most abundant structures for both diastereomers presented in Figure 8 and Figure 9 show that both compounds are stacking in a similar mode, as confirmed by the very similar distance between the plane of the base pair and the camptothecin core given in Table 8.

The most important conformational features of both diastereomers are the chiral centers at C5 and C20, which give some stiffness and quite different overall shapes of molecules, with both substituents on the same, as in **1**, or on the opposite, as in **2**, sides of a plane of the camptothecin core. Figure 8 and Figure 9 show a comparison of the most populated structures of both diastereomers, which were modeled and confronted with the experimental results of titration in the ^1^H NMR spectra. Both structures have ring A stacking a ring of cytidine C8, and ring C stacking the pyrimidine ring of guanosine G1.

The distances between the chosen protons given in Table 8 indicate the nearly parallel stacking of the components in a complex, and similar distances between interacting stacking molecules in both diastereomers. The complex models are compatible with the experimental NMR results and allow rational interpretation of several observed phenomena.

## 3. Materials and Methods

### 3.1. Chemical Substrates

The octamer duplex d(GCGATCGC)_2_ **3** was purchased from Integrated DNA Technologies and purified by filtering on a membrane of 3 kDa. The compounds **1** (5(*R*)-hydroxymethyl-7-ethyl-9-(N-azetidinyl)methyl-10-hydroxycamptothecin hydrochlorides) and **2** (5(*S*)-hydroxymethyl-7-ethyl-9-(N-azetidinyl)methyl-10-hydroxy-camptothecin hydrochlorides) were synthesized and purified as described earlier [14].

### 3.2. HPLC Analysis

HPLC was performed using an HPLC system from Shimadzu USA Manufacturing Inc. (Canby, OR, USA) consisting of a low-pressure gradient flow LC-20AT pump, a DGU-20A online solvent degasser, an SPD-M20A photodiode array detector, an SIL-10AF sample injector, and an FRC-10A fraction collector. Data were monitored using a Shimadzu LabSolution system.

#### 3.2.1. HPLC Separation and Purification

The compounds were separated from reaction mixture using HPLC with a Phenomenex Gemini 5 µm NX-C18 110 Å 250 × 10 mm column using a mobile phase system of CH_3_CN/aqueous 0.1% HCOOH at the flow rate of 3 mL/min using the following gradient: 15% CH_3_CN for 7 min, to 20% CH_3_CN at 10 min, and to 50% CH_3_CN at 26 min. The course of the chromatography was monitored using UV detection at a wavelength of 260 nm. Fractions were collected and lyophilized. The products were converted to their corresponding hydrochlorides salts using 0.5% aqueous HCl (3 mL) and lyophilized.

#### 3.2.2. Compounds Purity

The purity of compounds was analyzed using HPLC with a Phenomenex Gemini 5 µm NX-C18 110 Å 250 × 4.6 mm column using a mobile phase system of CH_3_CN/aqueous 0.1% HCOOH at the flow rate of 1 mL/min using the following gradient: 12% CH_3_CN to 15% CH_3_CN at 7 min, 15% CH_3_CN for 10 min, and to 50% CH_3_CN at 22 min. The course of the chromatography was monitored using UV detection at a wavelength of 260 nm.

#### 3.2.3. HPLC Analysis of Incubation Samples

The incubation samples were analyzed using HPLC with a Phenomenex Gemini 5 µm NX-C18 110 Å 250 × 4.6 mm column using a mobile phase system of CH_3_CN/aqueous 0.1% HCOOH at the flow rate of 1 mL/min using the following gradient: 12% CH3CN to 20% CH3CN at 17 min, and to 50% CH_3_CN at 22 min. The course of the chromatography was monitored using UV detection at a wavelength of 260 nm.

### 3.3. Sample Preparation

Samples of **1** and **2** for the stability experiments (the ^1^H, ^13^C NMR spectra and DOSY measurements) were prepared in D_2_O with internal standard TSPA-*d4,* pH 5. The spectra were recorded at 25 °C.

The other measurements (aggregation of **1** and **2** and binding of **1** and **2** with **3**) were performed in D_2_O or H_2_O/D_2_O (90%/10%) buffer (25 mM NaCl/25 mM K_3_PO_4_), with internal standard TSPA-*d4*, at 10 °C. To avoid formation of the carboxylate form of compounds **1** and **2** in buffer, the measurements were performed at pH 6.4.

### 3.4. NMR Experiments

The NMR spectra were recorded at 283 or 298 K using a Varian VNMRS-500 spectrometer (Varian, Inc., NMR Systems, Palo Alto, CA, USA), operated at 499.8 and 125.7 MHz for ^1^H and ^13^C measurements, respectively. All experiments were run using the standard Varian software (VnmrJ version 3.1A software from Varian, Inc., NMR Systems, Palo Alto, CA, USA). The spectrometer was equipped with an inverse ^1^H{^31^P–^15^N} 5 mm Z-SPEC Nalorac IDG500-5HT probe (Nalorac Corp., Martinez, CA, USA) with an actively shielded *z*-gradient coil, to give a maximum gradient strength of 61.1 G cm^−1^.

The assignment of proton resonances was confirmed with the aid of proton–proton coupling patterns. The assignment of carbon atoms was undertaken based on the ^1^H–^13^C HSQC (heteronuclear single quantum correlation) and ^1^H–^13^C HMBC (heteronuclear multiple bond correlation) experiments (parameters see below).

The NMR spectra were referenced using sodium 3-trimethylsilyltetradeuteriopropionate, TSPA-*d*_4_, as an internal reference. The concentrations of **1**, **2**, **3** in the tested solutions were determined against quantitatively added TSPA-*d*_4_. One-dimensional proton spectra were acquired in conditions that assured quantitative measurements using 256–2048 scans (depending on the concentration), with a 30° pulse width and a relaxation delay of 10 s.

The experiments were performed in the following conditions:

1D ^1^H NMR spectra: spectral width 8000 Hz, 16–256 scans, 32 K complex points, acquisition time 2 s, relaxation delay 2 s.
NOESY: spectral widths 5000 Hz in both dimensions, 1024 complex points in *t*_2_, 512 complex points in *t*_1_, 64 scans per increment, relaxation delay 1 s, and mixing time 200 ms.
^1^H-^13^C HSQC: spectral widths 6000 Hz in F2 and 21,600 Hz in F1, 1024 complex points in *t*_2_, 400 complex points in *t*_1_, 64 scans per increment, relaxation delay 1s, ^1^*J*(C,H) = 146 Hz.
^1^H-^13^C HMBC: spectral widths 6000 Hz in F2 and 23,880 Hz in F1, 1024 complex points in *t*_2_, 400 complex points in *t*_1_, 256 scans per increment, relaxation delay 1 s, ^n^*J*(C,H) = 5 or 8 Hz.

The data were processed with linear prediction in (when necessary) *t*_1_, followed by zero-filling in both dimensions. Gaussian weighting functions were applied in both domains prior to Fourier transformation.

Oneshot [17] DOSY spectra: 128–832 transients, 16 dummy scans, diffusion time (Δ) 180 ms, total diffusion encoding gradient duration (δ) 2 ms for **1** or **2** and 2.6 ms for octane **3** and bound species, 16 values of the diffusion-encoding gradients incremented from 6 to 50 G/cm in such steps that the strength of the next gradient was equal to the previous gradient squared. Other parameters include the following: a sweep width of 8000 Hz, 32 k data points, an acquisition time of 2.0 s, and a relaxation delay of 2.0 s. Processing was carried out using the VARIAN VNMRJ software, with the option of correction for spatially nonuniform pulsed field gradients. For these measurements, 5 mm sapphire NMR tubes were used to eliminate convection artefacts.

### 3.5. Calculating Self-Association Constants of ***1*** and ***2***

The concentration dependence of chemical shifts of **1** or **2** protons signals was obtained from NMR experiment. A 1.2 mM solution of **1** and a 0.77 mM solution of **2** in phosphate buffer (D_2_O, 25 mM NaCl/25 mM K_3_PO_4_) at pH 6.4 were diluted stepwise with the same buffer down to about 0.003 mM. The one dimensional ^1^H NMR spectra were measured for these solutions in conditions assuring the quantitative measurements of a concentration. The concentration in the solutions was measured by comparing the integral of chosen proton signals of **1** or **2** with the integral of TSPA-*d*_4_ signal. The experimental data were used for evaluation of the association constant K_a_ of **1** or **2** (L) by the isodesmic model [18], which assumes that L associates to form stacks, with a single self-association constant K_a_ The data from the dilution experiment was used to fit Equation (1) [19].

(1) $$\Delta \delta_{\mathrm{obs}}=\Delta \delta_{\max}\;\cdot {\;K}_{a}\cdot \;\left[L_{0}\right]\cdot \;{\left(\frac{2}{1+\sqrt{4\;\cdot {\;K}_{a}\cdot \;\left[L_{0}\right]+1}}\right)}^{2}$$

where Δδ_obs_ = δ_mon_ − δ_obs_ means the change in the observed average chemical shift for protons signals of L at different concentrations, δ_obs_ means the observed average chemical shifts of protons signals of L at different concentrations, δ_mon_ means the chemical shifts of protons signals of L at infinitely low concentration (in monomer form), Δδ_max_ refers to the maximal chemical shift change between monomer and oligomer, [L_0_] means the total concentrations of L, K_a_ means the association constant.

### 3.6. Calculating Binding Constants Based on ^1^H NMR Titration

The binding constants (K_a_) of the complexes DNA·L can be calculated based on observed chemical shifts changes during titration of L with DNA. Assuming that the binding equilibrium in the NMR experiment is established very quickly (dynamic-equilibrium method), and complexes exist with a stoichiometry of 1:1 the binding behavior can be described with the following mathematical model:

(2) $$\begin{array}{c} \mathrm{DNA}+L\;↔\mathrm{DNA}\cdot L\\ K_{a}=\frac{\left[\mathrm{DNA}\cdot L\right]}{\left[\mathrm{DNA}\right]\left[L\right]} \end{array}$$

where L is compound **1** or **2**; DNA is octamer **3**; DNA·L is a 1:1 complex; [L], [DNA] and [DNA·L] are the equilibrium concentrations of, L, DNA and DNA·L complex, respectively; K_a_ is the corresponding binding constants.

The NMR chemical shift changes observed during titration of the compound **1** or **2** by octamer **3** were fitted by the non-linear least-square method by using the following Equation (3) [20]:

(3) $$\Delta \delta =\delta_{\mathrm{obs}}-\delta_{L}=\frac{{\Delta \delta }_{\mathrm{DNA}\cdot L}K_{a}\left[\mathrm{DNA}\right]}{\left(1+K_{a}\left[\mathrm{DNA}\right]\right)}$$

where: Δδ is the chemical shifts change of the proton’s signals of L in the presence of DNA at different concentrations, δ_obs_ is the observed average chemical shifts of the protons signals of L at different concentrations of DNA, δ_L_ is the chemical shifts of the protons signals of L in the absence of DNA, Δδ_DNA_·_L_ is the maximal chemical shifts change of protons signals of L in the complex DNA·L in relation to unbound L.

### 3.7. Calculating Binding Constants from the Diffusion Coefficients

The binding constants (K_a_) of the complexes were also estimated by the analysis of the diffusion coefficient of octamer **3** (DNA), compounds **1** or **2** (L), and DNA·L complex as a function of the DNA and L concentration [21] according to Equation (2). In the case in which the exchange rate between the uncomplexed and complexed species is fast on the NMR timescale, the observed diffusion coefficients (D, (m^2^ s^−1^)) are a weighted average of the diffusion coefficients of the uncomplexed and complexed species, where the weighting factors are the relative population sizes of the respective species. Thus, the observed diffusion coefficients may be expressed as:

(4) $$D_{\mathrm{OBS}-L}={\mathrm{MF}}_{L}D_{L}+\left(1-{\mathrm{MF}}_{L}\right)D_{\left[\mathrm{DNA}\cdot L\right]}$$

(5) $$D_{\mathrm{OBS}-\mathrm{DNA}}={\mathrm{MF}}_{\mathrm{DNA}}D_{\mathrm{DNA}}+\left(1-{\mathrm{MF}}_{\mathrm{DNA}}\right)D_{\left[\mathrm{DNA}\cdot L\right]}$$

where D_OBS-L_ and D_OBS-DNA_ are the observed averaged diffusion coefficients for L and DNA measured in the solution containing both L and DNA; D_L_ and D_DNA_ are the diffusion coefficients for uncomplexed L and uncomplexed DNA; MF_L_ and MF_DNA_ are the molar fractions of uncomplexed L and uncomplexed DNA in the solution containing both molecules; and D_[DNA·L]_ is the diffusion coefficient for the DNA·L complex.

The K_a_ can be also expressed as:

(6) $$K_{a}=\frac{\left[\mathrm{DNA}\cdot L\right]}{\left(C_{\mathrm{DNA}}-\left[\mathrm{DNA}\cdot L\right]\right)\left(C_{L}-\left[\mathrm{DNA}\cdot L\right]\right)}\;$$

where: C_DNA_ and C_L_ are the initial concentrations of DNA and L. The unknown complex concentration can be calculated from equations:

(7) $$\left[\mathrm{DNA}\cdot L\right]=\left(1-{\mathrm{MF}}_{\mathrm{DNA}}\right)C_{\mathrm{DNA}}$$

(8) $$\left[\mathrm{DNA}\cdot L\right]=\left(1-{\mathrm{MF}}_{L}\right)C_{L}$$

In the case where the DNA molecule is much larger than the L, it can be assumed that the diffusion coefficient of the DNA·L complex is the same as that of the DNA molecule:

(9) $$\;D_{\left[\mathrm{DNA}\cdot L\right]}≅D_{\mathrm{OBS}-\mathrm{DNA}}$$

By combining Equations (4), (6), (8) and (9), K_a_ can be determined.

This formal treatment of the data includes a simplification which may cause that the results are affected by error.

### 3.8. Molecular Dynamics Calculations

All molecular mechanical calculations were carried out by using the AMBER 14 suite of programs [22]. The starting structure of DNA octamer were build using NAB (Nucleic Acid Builder) being part of AMBER 14 package (AmberTools). The DNA was built as a complementary double-stranded AB helix (the average of A and B DNA canonical forms) with the d(GCGATCGC)_2_ sequence. To the boundary nucleic bases GC pair, compounds **1** and **2** were manually docked in all four possible stacking orientations. Additionally, the four starting orientations of the two bulky substituent’s (9-CH_2_-AZT and 7-Et) relative to the rest of SN38 compound and two stereoisomers (*R*) and (*S*) on carbon 5 of SN38 were taken into consideration. This gives a total of 32 systems, which were then subjected to molecular dynamics simulations (MD) as separate trajectories. The mixed ff12SB for DNA and GAFF force fields were used. The missing GAFF force field parameters were obtained using the antechamber and parmchk modules (AmberTools). The electrostatic potential (ESP) charges were obtained for SN38 derivative by the HF/6-31G* calculations using Gaussian09 program [23]. Next, the RESP charges were calculated by charge fitting with the multi-conformational procedure of the antechamber. Each complex was neutralized by adding Na+ cations, and next solvated by TIP3 water molecules with a spacing distance of about 12 Å around the system surface creating a periodic box. All complexes were subjected to molecular dynamics simulations (md) using pmemd.cuda Amber 14 module with the NVIDIA GPU acceleration. The particle mesh Ewald (PME) method was used to treat long-range electrostatic interactions a 10 Å cutoff was applied to the nonbonded Lennard–Jones interactions. The SHAKE algorithm was applied to constrain all bonds involving hydrogen atoms and a 2 fs time step was used in the dynamics simulation. First, the systems were minimized in two stages: the first stage restrains the atomic positions of the solute and only relaxes the water, and the second stage releases the restraint and allows all atoms to relax (both with 10,000 minimization steps). Next, the systems were slowly heated to 300K using NVT ensemble and 1,000,000 steps with the Langevin dynamics temperature control (gamma_ln = 1.0). Next, the systems were carefully equilibrated at NPT ensemble simulations at 1 bar pressure with gamma_ln = 5.0. The equilibrations last until the system reaches a converged density value usually for 10–20 ns. Finally, the NPT production molecular dynamics were run for each of 32 trajectories for 500 ns of simulations. The trajectories were combined separately for (*R*) and (*S*) SN38 stereoisomers and were used for further calculations and analysis.

#### 3.8.1. Calculating Binding Free Energies (Enthalpies) Using MM-PBSA and MM-GBSA Methods 

The combined MD trajectories were uniformly sampled, giving 400,000 structures for each of **1** and **2**. The water and Na^+^ cations were stripped, and the binding free energies (enthalpies) were calculated using MM-PBSA and MM-GBSA methods [24] according to the following equations:

ΔG°_Bind,Solv_ = ΔG°_Bind,Vacuum_ + ΔG°_Solv,Complex_ − (ΔG°_Solv,Ligand_ + ΔG°_Solv,Receptor_) (10)

Solvation free energies were calculated by either solving the linearized Poisson–Boltzmann or generalized Born equation for each of the three states (this provides the electrostatic contribution to the solvation free energy) and adding an empirical term for hydrophobic contributions:

ΔG°_Solv_ = ΔG°_electrostatic,__ϵ=80_ − ΔG°_electrostatic,__ϵ=1_ + ΔG°_hydrophobic_ (11)

ΔG°_Vacuum_ was obtained by calculating the average interaction energy between receptor and ligand, and taking the entropy change upon binding into account.

ΔG°_Vacuum_ = ΔE°_MM_ − TΔS° (12)

where: G°_Bind,Solv_ is the free energy of binding of solvated molecules; G°_Bind,Vacuum_ is the binding free energy in vacuum; G°_Solv,Complex_, G°_Solv,Ligand_, and G°_Solv,Receptor_ are the solvation free energy for complex, ligand, and receptor molecules; G°_Electrostatic_ is the electrostatic solvation free energy; G°_Hydrophobic_ is the hydrophobic (nonpolar) solvation free energy; E°_MM_ is the molecular mechanic energy; T is the temperature; and S° is the entropy.

The entropy contribution in our calculations was neglected because of comparison of states of similar entropy. All free energy calculations were carried out using the mm_pbsa.pl script from AmberTools.

#### 3.8.2. PM7 Semi-Empirical Calculations

The combined MD trajectories were uniformly sampled to give 40,000 structures for each of **1** and **2**. The water and Na^+^ cations were striped, and the DNA was shortened to one GC base pair complexed with an SN38 derivative. The remaining DNA base pair was capped from the cut side with the phosphorane groups. The structures thus prepared were energy minimized with the PM7 method, using the MOPAC2016 program [25]. The water solvent was approximated with the COSMO model [26].

#### 3.8.3. Cluster Analysis

The CPPTRAJ module implemented in the Amber package was used for cluster analysis. During cluster analysis, the similar conformations were identified and grouped. The cluster analyses were performed for structures used in PBSA/GBSA calculations and for structures from PM7 energy minimizations. During clustering analysis, the kmeans clustering algorithm was used. The RMSD of heavy atoms was used as distance metric calculated only for SN38 derivative and the neighboring two DNA base pairs for the structures from PBSA/GBSA calculations and one DNA base pair for structures from PM7 calculations. The clustering procedure was repeated several times and each time the low populated strange structures were gradually removed. Finally, for each system, several clusters have been obtained. For each cluster, the average energies were calculated (PBSA/GBSA or PM7) and the most representative’s structures were determined.

## 4. Conclusions

In the presented research, we have undertaken the characterization of novel nontoxic derivatives of SN38, potential Topo I inhibitors with favorable anti-neoplastic cytotoxicity against several cancer cells, with the aim of establishing, using NMR, their physicochemical properties in solution, which can be linked to their differential pharmacological properties.

The dilution isotherms appeared quite different for the studied diastereomers. Monitoring the chemical shift changes of proton signals during the dilution experiment allowed calculation of their self-association constant K_a_, which were 1.71 and 0.70 mM^−1^ for diastereomers **1** and **2**, respectively. The above experiments are relevant to an important pharmacology parameter, namely solubility in physiological conditions. The observed better solubility of diastereomer 1 vs. diastereomer 2 can be attributed to its enhanced self-association via stacking, which results in covering the hydrophobic core with a hydrophilic medium.

The property of chemical and stereochemical stability in water is crucial for a potential medicine that must reach its biological target in an unchanged form. Both diastereomers were therefore incubated in water solution at pH 5 to monitor the process of degradation by ^1^H and ^13^C NMR. The acquired spectra confirmed slow continuous retro Mannich elimination of a substituent at C9 with a half-life time t_½_ of ca. 50 days for both diastereomers, nevertheless the compounds were relatively stable within the first few days. The HPLC analysis provided evidence for immutability of the absolute configuration of the substituent at C5 based on comparison of the different retention times of the products after incubation in both cases.

The studied compounds are potential Topo I inhibitors; therefore, their interaction with a target DNA is of primary interest. Because there is a common consensus that Topo I inhibitors require a G-C base pair in a nicked DNA target [15], we deliberately chose a self-complementary model DNA oligomer with a G-C base pair at both ends, mimicking the G-C face in a nick of wild type DNA. Diastereomer **1** was much more strongly bound to d(GCGATCGC)_2_ than diastereomer **2**, with a K_a_ of 5.9 (5.00 ± 2.02) and 2.0 (3.76 ± 1.19) mM^−1^, respectively, which was concluded from the diffusion experiment and iterative simulation of bonding isotherms based on chemical shift changes during titration experiments of diastereomers with octamer **3** (in brackets). The molecular modeling suggested greater flexibility and diversity of bonding of diastereomer **2** to the ultimate G-C base pair, in concordance with its weaker bonding, although the most populated clusters of conformations are dominated by very similar geometry of both depicted molecular complexes.

Finally, it can be stated that results seem to be in rational agreement with the in vitro assay investigations showing slightly higher cytotoxic activity for diastereomer **2** (IC_50_ equal to 0.33 µmol/L for **1** and 0.11 µmol/L for **2** as determined using the HL-60 cell line). Recalling the well-established fact that only an inhibitor in monomer state can access a nick of DNA, better biotarget availability is expected for diastereomer **2** than for diastereomer **1**, due to relatively strong self-associations of **1** hindering other interactions. Furthermore, the conformational diversity of diastereomer **2** can allow the optimal geometry to be easily formed inside the nick of a complex, as compared with diastereomer **1** which, despite forming stronger bonds, has a less flexible definite conformation.

## Figures and Tables

**Table 1:** The chemical shift changes, Δδ ^1^ [Hz], of the proton signals of **1** and **2** in D_2_O buffer (25 mM NaCl/25 mM K_3_PO_4_, TSPA-*d_4_*) for different concentrations c [mM], pH 6.4, temp. 10 °C. The isodesmic association constants K_a_ [mM^−1^].

**Δδ for 1**												
**c_1_**	**Ring A**		**Ring B**		**Ring C**			**Ring D**	**Ring E**			
	**H12**	**H11**	**H23**		**H5**			**H14**	**H17a**	**H17b**	**H18**	**H19**
0.005	(8.15) ^2^	(7.60)	(1.26)		(6.16)			(7.59)	(5.65)	(5.50)	(2.05)	(1.02)
0.05	23.6	11.5	−8.7		10.6			19.7	5.3	4.2	3.8	2.2
0.09	39.8	20.6	−15.1		17.9			33.4	9.6	7.9	7.6	4.9
0.29	96.1	47.2	−35.8		37.7			78.7	23.6	18.8	17.4	11.5
0.65	142.0	69.5	−54.7		53.8			117.1	34.6	27.4	25.1	16.4
1.20	180.3	88.1	−71.5		67.2			150.1	44.1	34.1	32.2	20.9
K_a_ Δδ_max_ ^3^	1.61 367.0	1.74 174.6	1.34 −153.4		2.28 121.9			1.59 305.7	1.64 89.4	1.76 67.9	1.70 64.3	1.76 41.3
the average value of K_a_			1.71 ± 0.24									
**Δδ for 2**												
**c_2_**	**Ring A**		**Ring B**		**Ring C**			**Ring D**	**Ring E**			
	**H12**	**H11**	**H24**	**H23**	**H51**	**H28**	**H28**	**H14**	**H17a**		**H17b**	**H18**
0.003	(8.15) ^2^	(7.60)	(5.10)	(1.26)	(6.20)	(4.81)	(4.35)	(7.57)	(5.66)		(5.46)	(2.02)
0.05	2.5	1.9	1.0	0	2.5	2.1	1.2	2.3	1.6		1.6	0.5
0.08	3.4	2.1	1.9	0	3.2	2.3	1.4	2.8	2.0		1.7	1.7
0.26	8.3	4.5	3.6	−2.8	8.1	4.2	2.7	7.3	3.3		3.2	2.7
0.43	11.5	5.3	4.4	−6.4	11.0	5.0	2.6	10.1	3.6		3.9	2.2
0.77	19.4	8.9	7.5	−8.4	18.6	8.6	4.9	17.6	7.1		8.0	5.4
K_a_ Δδ_max_ ^3^	0.40 98.0	0.91 27.7	0.68 26.9	0.20 −67.3	0.42 89.4	0.82 27.7	1.03 14.8	0.35 96.5	1.53 15.7		0.41 40.9	0.98 15.1
the average value of K_a_			0.70 ± 0.38									

^1^ Δδ = δ *_at lowest concentration_* − δ*_at given concentration_*; positive values correspond to a low frequency shift, [Hz]; ^2^ in brackets, the chemical shifts of proton signals are given, δ [ppm]; ^3^ Δδ_max_—calculated maximum chemical shift changes related to the association process.

**Table 2:** The diffusion coefficients, Di × 10^−10^ [m^2^s^−1^] for different concentrations c [mM] for the studied diastereomers **1** and **2** in D_2_O buffer (25 mM NaCl/25 mM K_3_PO_4_), pH 6.4, temp. 10 °C.

1		2	
c [mm]	Di × 10^−10^ [m^2^s^−1^]	c [mm]	Di × 10^−10^ [m^2^s^−1^]
1.5	1.92 ± 0.05	0.77	2.18 ± 0.05
0.29	2.13 ± 0.10	0.19	2.22 ± 0.10
0.23	2.13 ± 0.10	0.13	2.24 ± 0.10

**Table 3:** The PGSE data for the binding of **1** or **2** with octamer **3** ^1^ in D_2_O buffer (25 mM NaCl/25 mM K_3_PO_4_), pH 6.4, temp. 10 °C.

	Concentration [mm]		D_OBS_	D_OBS-3_	MF_comp_	K_a_ ± 0.3
	c	c_3_	× 10^−10^ [m^2^ s^−1^]	× 10^−10^ [m^2^ s^−1^]		[mm^−1^]
**1**	0.29	0.92	1.11 ± 0.05	0.86 ± 0.05	0.80 ± 0.05	5.9
	0.45	0.92	1.14 ± 0.05	0.91 ± 0.05	0.81 ± 0.05	7.7
	1.89	1.26	1.34 ± 0.05	0.88 ± 0.05	0.55 ± 0.05	5.5
**2**	0.29	0.28	1.83 ± 0.10	0.93 ± 0.10	0.29 ± 0.05	2.1
	0.29	0.42	1.67 ± 0.10	0.92 ± 0.10	0.41 ± 0.05	2.4
	0.29	0.87	1.45 ± 0.10	0.92 ± 0.10	0.59 ± 0.05	2.0
	0.9	1.47	1.35 ± 0.10	0.84 ± 0.10	0.63 ± 0.05	1.8

^1^**c** and **c_3_** are the concentration of **1** or **2** and **3**, respectively; D_OBS_ is the measured diffusion coefficient for **1** or **2** in the presence of **3**; D_OBS-**3**_ is the measured diffusion coefficient for **3** in the presence of **1** or **2**; MF_comp_ is the calculated molar fraction of **1**–**3** or **2**–**3** complexes; K_a_ is a binding constant. The measured diffusion coefficients for uncomplexed species of **1** and **2** see Table 2; for **3** (0.77 mM) is equal 0.88 ± 0.05 × 10^−10^ [m^2^ s^−1^].

**Table 4:** The chemical shift changes, Δδ ^1^ [Hz], of proton signals of **1** or **2** in D_2_O buffer, pH 6.0 (25 mM NaCl/25 mM K_3_PO_4_), induced by interaction with octamer **3** in solutions of different concentrations of octamer **3**, c**_3_** [mM], and a constant concentration of **1** or **2,** c**_1_** = c**_2_** = 0.29 mM, temp. 10 °C. The binding constants, K_a_ [mM^−1^], were calculated based on chemical shift changes.

**Δδ for 1**										
**c_3_**	**Ring A**		**Ring B**	**Ring C**		**Ring D**	**Ring E**			
	**H12**	**H11**	**H22**	**H5**	**H28**	**H14**	**H17a**	**H17b**	**H18**	**H19**
0	(7.95) ^2^	(7.51)	(3.36)	(6.10)	(4.76)	(7.44)	5.60	(5.46)	(2.01)	(0.99)
0.02	3.9	6.6	10.1	11.2	5.0	2.6	4.3	4.3	3.6	2.4
0.06	6.2	13.5	19.3	28.3	12.1	4.7	8.7	10.9	8.2	5.5
0.12	13.2	26.8	31.3	56.5	23.0	9.8	17.5	21.6	16.2	10.7
0.25	23.4	42.1	49.3	89.2	36.9	18.3	30.7	35.1	26.8	18.1
0.56	46.2	69.2	80.7	143.5	60.4	35.4	51.2	57.2	44.1	30.1
0.87	59.0	82.7	93.2	-	71.1	44.2	60.0	67.6	52.9	35.6
K_a_ Δδ_max_^3^	1.37 ^4^ 118.8	5.34 105.7	7.57 111.4	7.72 197.6	5.89 89.4	1.54 ^4^ 85.0	4.76 79.4	5.56 85.7	5.38 66.9	4.89 47.0
the average value of K_a_					5.00 ± 2.02 (5.89 ± 1.07) ^4^					
**Δδ for 2**										
**c_4_**	**Ring A**		**Ring B**		**Ring C**		**Ring D**	**Ring E**		
	**H12**	**H11**	**H22**	**H23**	**H5**	**H28**	**H14**	**H17a**	**H17b**	**H19**
0.00	(8.16) ^2^	(7.62)	(3.37)	(1.27)	(6.19)	(4.81)	(7.57)	(5.65)	(5.46)	(0.99)
0.02	5.7	4.1	6.7	1.4	7.3	2.8	4.3	2.1	1.5	0
0.07	17.3	12.9	15.5	3.4	22.7	9.1	13.6	6.1	4.1	−0.9
0.13	31.1	23.4	28.7	5.5	40.3	15.8	24.3	10.4	7.0	−1.7
0.28	60.2	45.4	57.3	9.6	76.7	30.0	46.8	21.3	13.2	−3.9
0.65	-	74.9	88.1	14.5	-	49.0	77.4	-	20.5	−6.5
1.13	123.5	-	110.9	18.4	153.0	62.5	96.2	-	-	−7.3
1.47	133.5	-	115.3	20.7	163.2	65.7	104.6	-	-	−7.0
K_a_ Δδ_max_^3^	3.11 167.5	3.52 119.9	3.93 138.0	3.86 23.8	3.64 199.3	3.40 89.4	3.09 130.4	1.66 ^5^ 81.2	6.48 ^5^ 28.0	4.88 −8.85
the average value of K_a_					3.76 ± 1.19					

^1^ Δδ = δ**_A_** − δ_obs_: the chemical shift change of the proton signals of **1** or **2**; δ**_A_** is the chemical shifts in proton signals of **1** or **2** in the absence of octamer **3**, *δ_obs_* is the observed average chemical shifts of the proton signals of **1** or **2** in the presence of octamer **3** at a given concentration. Positive values correspond to a low frequency shift, [Hz]. ^2^ in brackets, the chemical shifts δ, [ppm], of proton signals of **1** or **2** at c**_1_** = c**_2_** = 0.29 mM, are given. ^3^ Δδ_max_: calculated maximum chemical shift changes related to the complexation process, [Hz]. ^4^ the larger value is obtained when omitting the calculations for the H12 and H14 protons. ^5^ the average value of K_a_ for two geminal protons is 4.07.

**Table 5:** The chemical shift changes, Δδ ^1^ [Hz], of proton signals of octamer **3** in D_2_O buffer (25 mM NaCl/25 mM K_3_PO_4_), pH 6.0, temp. 10 °C, upon interaction with **1** or **2** at a concentration of octamer **3** equal to 0.02 mM and a concentration of **1** or **2** equal to 0.29 mM.

Δδ										
	G1-H8	G1-H1′	G1-H5′	C2-H6	C2-H5	A4-H8	A4-H2	A4-H1′	T5-H6	C8-H1′
	(7.98) ^2^	(5.99)	(3.72)	(7.42)	(5.38)	(8.24)	(7.79)	(6.28)	(7.19)	(6.19)
**1**	60.2	36.4	−28.8	20.3	28.3	11.4	10.5	9.9	10.3	31.9
**2**	11.3	26.4	−16.3	5.0	9.6	1.4	1.4	3.0	1.9	3.5

^1^ Δδ = δ**_B_** − δ_obs_ is the chemical shift change in the proton signals of **3**, δ**_B_** is the chemical shifts of the proton signals of **3** in the absence of **1** or **2**, *δ_obs_* is the observed average chemical shifts of the proton signals of **3** in the presence of **1** or **2**. Positive values correspond to a low frequency shift, [Hz]; protons marked in red form the base pair in a self-complementary octamer **3**, interacting with **1** or **2** with great relevance. ^2^ in brackets, the chemical shifts of the proton signals of octamer **3** in D_2_O buffer, (25 mM NaCl/25 mM K_3_PO_4_, TSPA-*d*_4_), pH 6.0, δ, [ppm].

**Table 6:** Diastereomer **1** C5(*R*), PM7 most populated cluster energies.

No.	Structure	PM7 ENERGY [kcal/mol]		% Population
		Clust. Average	10% Clust. Min.	
1	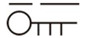	−1174.25 ± 3.79	−1181.15	20.39
2	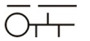	−1179.37 ± 3.39	−1185.18	17.14
3	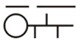	−1177.11 ± 3.94	−1184.58	14.25

**Table 7:** Diastereomer **2** C5(*S*), PM7 clusters energies.

No.	Structure	PM7 ENERGY [kcal/mol]		% Population
		Clust. Average	10% Clust. Min.	
1	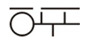	−1178.76 ± 4.77	−1186.83	20.19
2	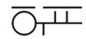	−1177.52 ± 4.07	−1183.82	16.28

**Table 8:** Internuclear distances in complexes of an ultimate G1-C8 base pair of octamer **3** and compounds **1** and **2**.

Atoms in		Distances [Å]			
Cytidine	**1**, **2**	**1** struct. no. 1 *	**2** struct. no. 1 #	**1** struct. no. 2 *	**2** struct. no. 2 #
C8-H5	H11	4.14	3.83	3.28	3.24
C8-H5	H12	4.69	3.87	3.05	5.18

* The same structure as in Table 6. # The same structure as in Table 7.

## Data Availability

The authors confirm that the data supporting the findings of this study are available within the article and its Supplementary Materials.

## Supplementary Materials

The following are available online at https://www.mdpi.com/article/10.3390/ijms22158190/s1. Figure S1: HPLC analysis of the crude reaction mixture. Figure S2: Atomic numbering and schematic representation of the relative positions of protons of **1**, **2**, **1A**, **2A**. Table S1: The experimental ^1^H and ^13^ C NMR chemical shifts for **1**, **1A** in D_2_O, pH 5, temp. 25 °C. Table S2: The experimental ^1^H and ^13^ C NMR chemical shifts for **2**, **2A** in D_2_O, pH 5, temp. 25 °C. Table S3: The experimental ^1^H and ^13^ C NMR chemical shifts for **2**, **2A** in buffer H_2_O/D_2_O, pH 6, temp. 25 °C. Table S4: The chemical shifts changes of the protons signals of **1** in D_2_O buffer, pH 6.4, temp. 10 °C and the isodesmic association constants K_a_. Table S5: The chemical shifts changes of the proton’s signals of **2** in D_2_O buffer, pH 6.4, temp. 10 °C and the isodesmic association constants K_a_. Table S6: The chemical shifts changes of proton signals of **1** in D_2_O buffer, pH 6 induced by interaction with octamer **3** in solutions of different concentrations of octamer **3**, and constant concentration of **1**; temp. 10 °C. The binding constants, K_a_ (mM^−1^) were calculated based on chemical shifts changes. Table S7: The chemical shifts changes of proton signals of **2** in D_2_O buffer, pH 6 induced by interaction with octamer **3** in solutions of different concentrations of octamer **3**, and constant concentration of **2**; temp. 10 °C. The binding constants, K_a_ (mM^−1^) were calculated based on chemical shifts changes. Table S8: Diastereomer **1** (*R*)C-5, PM7 clusters energies. Table S9: Diastereomer **2** (*S*)C-5, PM7 clusters energies. Table S10: Diastereomer **1** (*R*)C-5, PBSA and GBSA clusters energies. Table S11: Diastereomer **2** (*S*)C-5, PBSA and GBSA clusters energies. Figure S3. Molecular model of MD of the less abundant structures of the complexes of the ultimate base pair G1-C8 of a self-complementary duplex d(GCGATCGC)_2_ with diastereomers **1** and **2**.
