# Supplementary material for: Novel Nontoxic 5,9-Disubstituted SN38 Derivatives: Characterization of Their Pharmacological Properties and Interactions with DNA Oligomers
Source: Int J Mol Sci. 2021 Jul 30;22(15):8190. doi: 10.3390/ijms22158190 (PMC8347432; doi:10.3390/ijms22158190)
Supplement: Supplementary file 1 [file ijms-22-08190-s001.zip › ijms-1290339-supplementary.pdf]

Supplementary Materials

Novel nontoxic 5,9-disubstituted SN38 derivatives as Topo I poisons: characterization of their pharmacological properties and interactions with DNA oligomers

Elżbieta Bednarek, [a]* Wojciech Bocian, [a] Magda Urbanowicz, [a] Jerzy Sitkowski, [a] Beata Naumczuk, [a,b] Lech Kozerski [a]

[1] Falsified Medicines and Medical Devices Department, National Medicines Institute, Chełmska 30/34, 00-725 Warsaw, Poland
[2] Institute of Organic Chemistry, Polish Academy of Sciences, Kasprzaka 44/52, 01-224 Warsaw, Poland;

*Correspondence: Elżbieta Bednarek: *e-mail: e.bednarek@nil.gov.pl*

National Medicines Institute 00-725 Warsaw, Chełmska 30/34

Synthesis of the hydrochloride of 5-(*R*)-Hydroxymethyl-7-ethyl-9-(N-azetidinyl)methyl-10-hydroxycamptothecin and its diastereomer 5-(*S*).

SN38 (7-ethyl-10-hydroxycamptothecin·H_2O) (4.46 mg; 10.9 × 10^{-3} mmol) was suspended in 3 mL of CH_3CN and then supplemented with 37% aqueous CH_2O (10.6 µL; 0.13 mmol) and 7.6 µL of 98% azetidine (0.13 mmol). The resulting mixture was stirred at 80°C. The reaction ended 30 min after the substrate dissolved. The solvent was evaporated under vacuum, and the residue was rinsed with diethyl ether (3 × 1 mL). The residue was purified using HPLC with a Phenomenex Gemini 5 µm NX-C18 110 Å 250 × 10 mm column using a mobile phase system of CH_3CN/aqueous 0.1% HCOOH at the flow rate of 3 mL/min using the following gradient: 15% CH_3CN for 7 min, to 20% CH_3CN at 10 min, and to 50% CH_3CN at 26 min. The course of the chromatography was monitored using UV detection at a wavelength of 260 nm (see Fig. 1S). Fractions were collected with retention times of **5.7 min (1'), 11.0 min (2'), 19.8 min (1A), 21.0 min (2A)** and were lyophilized. The products were converted to their corresponding hydrochlorides salts using 0.5% aqueous HCl (3 mL) and lyophilized.

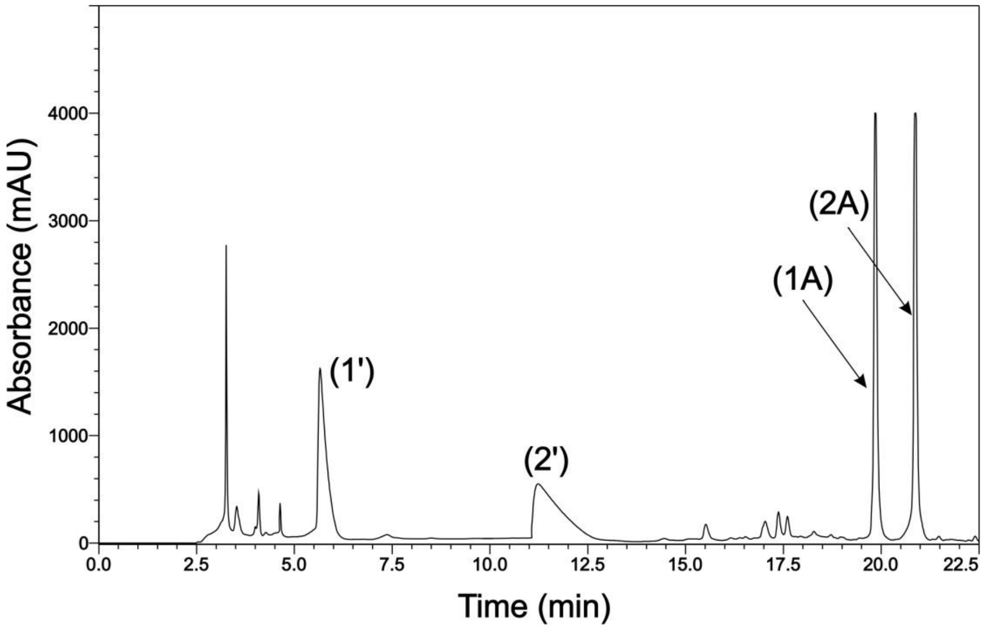

Figure S1. HPLC analysis of the crude reaction mixture showing the following compounds: 5-(*R*)-Hydroxymethyl-7-ethyl-9-(N-azetidinyl)methyl-10-hydroxycamptothecin **1'** and its diastereomer 5-(*S*) **2'** as formates and main side products: **1A** and **2A** – as results of retro Mannich transformation in position C9 of compounds **1'** and **2'**, respectively. HPLC was performed on Shimadzu USA Manufacturing Inc. (Canby, OR, USA), consisted of a low-pressure gradient flow LC-20AT pump, a DGU-20A on-line solvent degasser, an SPD-M20A photodiode array detector, an SIL-10AF sample injector, and an FRC-10A fraction collector. Data were monitored using the Shimadzu LabSolution system.

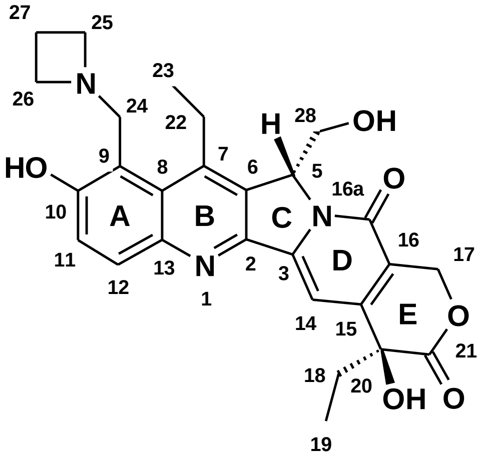

1 5-(*R*)-Hydroxymethyl-7-ethyl-9-(N-azetidinyl)methyl-10-hydroxycamptothecin

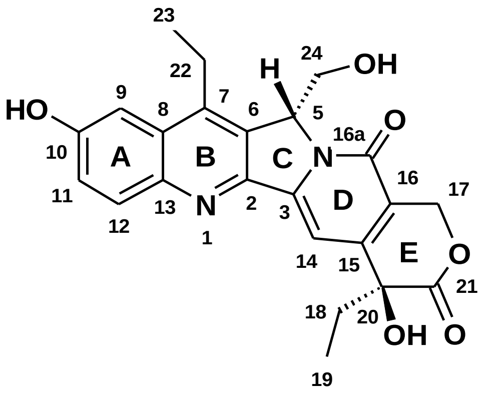

1A 5-(*R*)-hydroxymethyl-7-ethyl-10-hydroxycamptothecin (main product of the retro Mannich transformation of **1**)

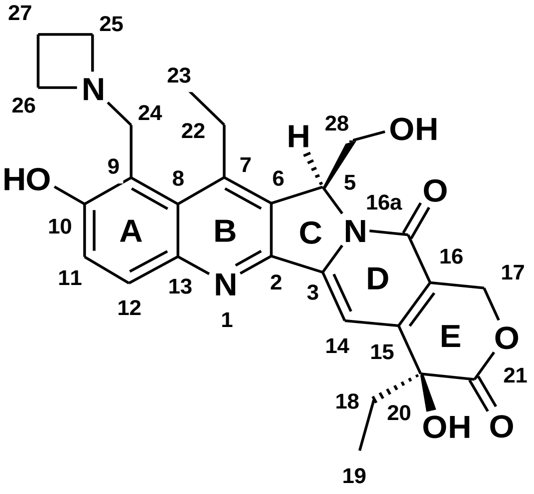

2 5-(*S*)-Hydroxymethyl-7-ethyl-9-(N-azetidinyl)methyl-10-hydroxycamptothecin

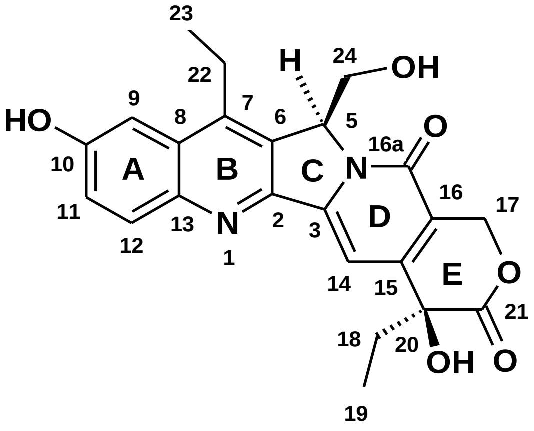

2A 5-(*S*)-hydroxymethyl-7-ethyl-10-hydroxycamptothecin (main product of the retro Mannich transformation of **2**)

Figure S2. Atomic numbering and schematic representation of the relative positions of protons of **1**, **2**, **1A** (main product of the retro Mannich transformation of **1** in position C9) and **2A** (main product of the retro Mannich transformation of **2** in position C9).

Table S1. The experimental ^{1}H and ^{13}C NMR chemical shifts δ [ppm] for **1, 1A** - main product of the retro Mannich transformation of **1** and azetidine in D_2O, pH 5, temp. 25 °C (ref. to TSPA-d_4).

**1**			**1A**		
Numbering of atom position (see figure 2S)	δ_{1H} *	δ_{13C} #	Numbering of atom position (see figure 2S)	δ_{1H} *	δ_{13C} #
**2** (C)	-	151.7 (*H5, H14*)	**2** (C)	-	151.0 (*H14*)
**3** (C)	-	149.2 (*H14*)	**3** (C)	-	149.1 (*H14*)
**5 (CH)**	**6.10 (*brs*, 1H)**	**68.3**	**5 (CH)**	**5.50 (*brs*, 1H)**	**67.6**
**6** (C)	-	134.4 (*H5, H22, H28*)	**6** (C)	-	130.5 (*H5, H22, H28*)
**7** (C)	-	147.9 (*H22, H23*)	**7** (C)	-	148.0 (*H22, H23*)
**8** (C)	-	130.6 (*H12, H22, H24*)	**8** (C)	-	131.3 (*H12, H22*)
**9 (C)**	**-**	**111.8 (*H11, H24*)**	**9 (CD)**	**-**	**108.6 (*H11*)**
**10** (C)	-	160.2 (*H12, H24*)	**10** (C)	-	158.3 (*H12*)
**11**(CH)	7.56 (*d*, 1H, *J* = 9.3)	124.8	**11**(CH)	7.31 (*d*, 1H, *J* = 9.1)	125.6
**12** (CH)	8.04 (*d*, 1H, *J* = 9.3)	136.3	**12** (CH)	7.76 (*d*, 1H, *J* = 9.1)	133.0
**13** (C)	-	148.1 (*H11*)	**13** (C)	-	146.0 (*H11*)
**14** (CH)	7.48 (*s*, 1H)	101.0	**14** (CH)	7.30 (*s*, 1H)	100.6
**15** (C)	-	153.7 (*H17, H18*)	**15** (C)	-	153.6 (*H17, H18*)
**16** (C)	-	122.3 (*H14, H17*)	**16** (C)	-	121.9 (*H14, H17*)
**16a** (N-**C**=O)	-	161.1 (*H17*)	**16a** (N-**C**=O)	-	160.7 (*H17*)
**17** (CH_2)	5.46 (*d*, 1H, *J* = 16.4)<br>5.59 (*d*, 1H, *J* = 16.4)	69.0	**17** (CH_2)	5.38 (*d*, 1H, *J* = 16.1)<br>5.52 (*d*, 1H, *J* = 16.1)	68.9
**18** (CH_2)	2.02 (*q*, 2H, *J* = 7.5)	33.5 (*H19*)	**18** (CH_2)	1.95 (*q*, 2H, *J* = 7.5)	33.6 (*H19*)
**19** (CH_3)	0.99 (*t*, 3H, *J* = 7.5)	9.9 (*H18*)	**19** (CH_3)	0.95 (*t*, 3H, *J* = 7.5)	9.9 (*H18*)
**20** (C)	-	76.5 (*H14, H18, H19*)	**20** (C)	-	76.5 (*H14, H18, H19*)
**21** (O-**C**=O)	-	177.9 (*H17, H18*)	**21** (O-**C**=O)	-	177.8 (*H17, H18*)
**22** (CH_2)	3.36 (*m*, 2H)	28.5 (*H23*)	**22** (CH_2)	3.16 (*m*, 1H); 2.99 (*m*, 1H)	25.0 (*H23*)
**23** (CH_3)	1.32 (*t*, 3H, *J* = 7.4)	16.7 (*H22*)	**23** (CH_3)	1.30 (*t*, 3H, *J* = 7.4)	16.3
**24** (CH_2)	5.04 (*d*, 1H, *J* = 14.7)<br>5.12 (*d*, 1H, *J* = 14.7)	53.7 (*H25, H26*)	**24** (CH_2)	4.08 (*dd*, 1H, *J* =12.4, 2.3)<br>4.65 (*dd*, 1H, *J* =12.4, 2.3)	60.9
**25** (CH_2)$	4.07 (*m*, 1H), 4.17 (*m*, 1H)	57.3 (*H24*)			
**26** (CH_2)	4. 08 (*m*, 1H), 4.24 (*m*, 1H)	57.6 (*H24*)			
**27** (CH_2)	2.40 (*m*, 1H); 2.50 (*m*, 1H)	18.9	azetidine	4.13 (*t*, 4H, *J* = 8.4)<br>2.55 (*quint*, 2H, *J* =8.4)	49.7<br>21.3
**28** (CH_2)	4.31 (*dd*, 1H, *J* =12.6, 2.2)<br>4.75 (*dd*, 1H, *J* =12.6, 2.2)	61.4			

* in brackets (multiplicity, number of protons, proton-proton coupling constants J_{HH} [Hz]);
\# in brackets the heteronuclear multiple bond diagnostic correlation between the given carbon atom and the showing proton(s) are presented;
$ the assignment in position **25** and **26** can be interchanged; *brs* broad singlet, *d* doublet, *dd* doublet of doublets, *m* multiplet, *s* singlet, *t* triplet, *q* quartet, *quint* quintet.

Table S2. The experimental ^{1}H and ^{13}C NMR chemical shifts δ [ppm] for **2, 2A** - main product of the retro Mannich transformation of **2** and azitidine in D_2O, pH 5, temp. 25 °C (ref. to TSPA-d_4).

**2**			**2A**		
Numbering of atom position (see figure 2S)	δ_{1H} *	δ_{13C} #	Numbering of atom position (see figure)	δ_{1H} *	δ_{13C} #
**2** (C)	-	151.9 (*H14*)	**2** (C)	-	151.5 (*H14*)
**3** (C)	-	149.4 (*H14*)	**3** (C)	-	149.5 (*H14*)
**5 (CH)**	**6.19 (*t*, 1H, *J* = 2.1)**	**68.3**	**5 (CH)**	**5.62 (*brs*, 1H)**	**67.5**
**6** (C)	-	134.4 (*H5*)	**6** (C)	-	130.7 (H22,*H28*)
**7** (C)	-	147.7 (*H22, H23*)	**7** (C)	-	148.0 (*H22, H23*)
**8** (C)	-	130.6 (*H12, H22, H24*)	**8** (C)	-	131.1 (*H22*)
**9 (C)**	-	**111.9 (*H11,H24*)**	**9 (CD)**	**-**	**n.o.**
**10** (C)	-	160.4 (*H24*)	**10** (C)	-	158.2 (*H12*)
**11**(CH)	7.63 (*d*, 1H, *J* = 9.2)	124.9	**11**(CH)	7.34 (*d*, 1H, *J* = 9.1)	125.3
**12** (CH)	8.17 (*d*, 1H, *J* = 9.2)	136.5	**12** (CH)	7.86 (*d*, 1H, *J* = 9.1)	133.1
**13** (C)	-	148.1 (*H11*)	**13** (C)	-	n.o.
**14** (CH)	7.57 (*s*, 1H)	101.4	**14** (CH)	7.47 (*s*, 1H)	100.7
**15** (C)	-	153.9 (*H17, H18*)	**15** (C)	-	153.8 (*H17, H18*)
**16** (C)	-	122.4 (*H14, H17*)	**16** (C)	-	122.3 (*H14, H17*)
**16a** (N-**C**=O)	-	161.4 (*H17*)	**16a** (N-**C**=O)	-	161.1 (*H17*)
**17** (CH_2)	5.45 (*d*, 1H, *J* = 16.2)<br>5.65 (*d*, 1H, *J* = 16.2)	69.1	**17** (CH_2)	5.49 (*d*, 1H, *J* = 16.1)<br>5.63 (*d*, 1H, *J* = 16.1)	69.0
**18** (CH_2)	2.02 (*m*, 2H)	33.4 (*H19*)	**18** (CH_2)	2.12 (*q*, 2H, *J* = 7.5)	33.5 (*H19*)
**19** (CH_3)	0.98 (*t*, 3H, *J* = 7.5)	10.0 (*H18*)	**19** (CH_3)	1.09 (*t*, 3H, *J* = 7.5)	10.2 (*H18*)
**20** (C)	-	76.5 (*H14, H18, H19*)	**20** (C)	-	76.5 (*H14, H18, H19*)
**21** (O-**C**=O)	-	177.9 (*H17, H18*)	**21** (O-**C**=O)	-	177.8 (*H17, H18*)
**22** (CH_2)	3.36 (*m*, 2H)	28.5 (*H23*)	**22** (CH_2)	2.99 (*m*, 1H); 3.19 (*m*, 1H)	25.0 (*H23*)
**23** (CH_3)	1.27 (*t*, 3H, *J* = 7.4)	16.5	**23** (CH_3)	1.30 (*t*, 3H, *J* = 7.4)	16.2 (*H22*)
**24** (CH_2)	5.07 (*d*, 1H, *J* = 14.3)<br>5.13 (*d*, 1H, *J* = 14.3)	53.7	**24** (CH_2)	4.13 (*m*, 1Hz)<br>4.72 (1H)$	60.7
**25, 26** (CH_2)	4.08 (*brs*, 2H)<br>4.21 (*m*, 2H)	57.5			
**27** (CH_2)	2.43 (*m*, 1H)<br>2.50 (*m*, 1H)	18.9	azetidine	4.14 (*t*, 4H, *J* = 8.4)<br>2.55 (*quint*, 2H, *J* =8.4)	49.6<br>21.3
**28** (CH_2)	4.34 (*dd*, 1H, *J* =12,7, 2.1)<br>4.81(1H)$	61.3			

* in brackets (multiplicity, number of protons, proton-proton coupling constants J_{HH} [Hz]);
\# in brackets the heteronuclear multiple bond diagnostic correlation between the given carbon atom and the showing proton(s) are presented;
$ the signal position was taken from HSQC experiment; n.o. - no observed;
brs broad singlet, *d* doublet, *dd* doublet of doublets, *m* multiplet, *s* singlet, *t* triplet, *q* quartet, *quint* quintet.

Table S3. The experimental ^{1}H and ^{13}C NMR chemical shifts δ [ppm] for **2** and **2A** main product of the retro Mannich transformation of **2** in buffer H_2O/D_2O, pH 6, temp. 25 °C (ref. to TSPA).

**2**			**2A**		
Numbering of atom position (see figure 2S)	δ_{1H} *	δ_{13C} #	Numbering of atom position (see figure)	δ_{1H} *	δ_{13C} #
**2** (C)	-	151.4 (*H14*)	**2** (C)	-	150.9 (*H14*)
**3** (C)	-	147.1 (*H14*)	**3** (C)	-	149.1 (*H14*)
**5 (CH)**	**6.15 (*brs*, 1H)**	**68.0**	**5 (CH)**	**5.45 (*brs*, 1H)**	**67.2**
**6** (C)	-	n.o	**6** (C)	-	130.2 (*H5, H22, H28*)
**7** (C)	-	n.o	**7** (C)	-	147.5 (*H9, H22, H23*)
**8** (C)	-	n.o	**8** (C)	-	130.9 (*H12, H22*)
**9 (C)**	**-**	**n.o**	**9 (CH)**	**7.18 (*d*, 1H, *J* = 2.3)**	**108.5**
**10** (C)	-	n.o	**10** (C)	-	158.1 (*H9, H12*)
**11**(CH)	7.57 (*d*, 1H, *J* = 9.3)	125.7	**11**(CH)	7.22 (*dd*, 1H, *J* = 9.1, 2.3)	125.1 (*H9*)
**12** (CH)	8.11 (*d*, 1H, *J* = 9.3)	135.9	**12** (CH)	7.72 (*d*, 1H, *J* = 9.1)	132.8
**13** (C)	-	n.o.	**13** (C)	-	145.6 (*H9,H11*)
**14** (CH)	7.53 (*s*, 1H)	104.0	**14** (CH)	7.42 (*s*, 1H)	100.4
**15** (C)	-	n.o.	**15** (C)	-	153.7 (*H17, H18*)
**16** (C)	-	n.o.	**16** (C)	-	122.0 (*H14, H17*)
**16a** (N-**C**=O)	-	n.o.	**16a** (N-**C**=O)	-	160.8 (*H17*)
**17** (CH_2)	5.44 (*d*, 1H, *J* = 16.3)<br>5.64 (*d*, 1H, *J* = 16.3)	68.8	**17** (CH_2)	5.50 (*d*, 1H, *J* = 16.1)<br>5.62 (*d*, 1H, *J* = 16.1	68.8
**18** (CH_2)	2.01 (*q*, 2H, *J* = 7.5)	33.3	**18** (CH_2)	2.14 (*q*, 2H, *J* = 7.5)	33.4 (*H19*)
**19** (CH_3)	0.98 (*t*, 3H, *J* = 7.5)	9.8	**19** (CH_3)	1.13 (*t*, 3H, *J* = 7.5)	10.1 (*H18*)
**20** (C)	-	n.o.	**20** (C)	-	76.3 (*H14, H18, H19*)
**21** (O-**C**=O)	-	n.o.	**21** (O-**C**=O)	-	177.4 (*H17, H18*)
**22** (CH_2)	3.34 (*m*, 2H)	28.2	**22** (CH_2)	2.90 (*m*, 1H)<br>3.11 (*m*, 1H)	24.8 (*H23*)
**23** (CH_3)	1.27 (*t*, 3H, *J* = 7.4)	16.3	**23** (CH_3)	1.26 (*t*, 3H, *J* = 7.4 Hz)	16.0 (*H22*)
**24** (CH_2)	5.03 (*d*, 1H, *J* = 14.3)<br>5.08 (*d*, 1H, *J* = 14.3)	53.9	**24** (CH_2)	4.07 (*dd*, 1H, *J* =12.4)<br>4.69 (1H)[$]	60.6
**25, 26** (CH_2)	4.12 (*brs*, 4H)	57.2			
**27** (CH_2)	2.43 (*brs*, 1H);<br>2.50 (*brs*, 1H)	18.7	azetidine	4.15 (*t*, 4H, *J* = 8.4)<br>2.55 (*quint*, 2H, *J* =8.4)	49.7<br>21.2
**28** (CH_2)	4.33 (*dd*, 1H, *J* =13.1)<br>n.o.	61.1			

[*] in brackets (multiplicity, number of protons, proton-proton coupling constants J_{HH} [Hz]);
[#] in brackets the heteronuclear multiple bond diagnostic correlation between the given carbon atom and the showing proton(s) are presented;
[$] the signal position was taken from HSQC experiment; n.o. no observed;
brs broad singlet, *d* doublet, *dd* doublet of doublets, *m* multiplet, *s* singlet, *t* triplet, *q* quartet, *quint* quintet.

Table S4. The chemical shifts changes, $\Delta\delta$ [1] [Hz], of the protons signals of **1** in D_2O buffer (25 mM NaCl/25 mM K_3PO_4, TSPA-d_4) for different concentration $\mathbf{c_1}$ [mM], pH 6.4, temp. 10 °C and the isodesmic association constants $\boldsymbol{K_a}$ [mM^{-1}].

Δδ										
	c_1	**ring A**		**ring B**	**ring C**	**ring D**	**ring E**			
		**H12**	**H11**	**H23**	**H5**	**H14**	**H17a**	**H17b**	**H18**	**H19**
1	0.005	(8.15)[2]	(7.60)	(1.26)	(6.16)	(7.59)	(5.65)	(5.50)	(2.05)	(1.02)
2	0.008	2.7	1.3	0	1.3	2.7	0.5	0	0	0
3	0.015	6.3	3.5	−3.3	3.8	6.0	1.7	0.8	0	0.6
4	0.028	14.1	7.7	−5.8	7.1	12.4	3.7	2.8	2.4	1.5
5	0.05	23.6	11.5	−8.7	10.6	19.7	5.3	4.2	3.8	2.2
6	0.09	39.8	20.6	−15.1	17.9	33.4	9.6	7.9	7.6	4.9
7	0.16	63.7	32.1	−24.2	27.6	52.8	15.6	12.4	11.8	7.7
8	0.29	96.1	47.2	−35.8	37.7	78.7	23.6	18.8	17.4	11.5
9	0.42	119.6	59.7	−42.8	46.4	98.7	30.3	23.7	22.0	14.6
10	0.65	142.0	69.5	−54.7	53.8	117.1	34.6	27.4	25.1	16.4
11	0.88	162.3	79.5	−62.8	61.3	134.7	39.7	30.8	28.8	18.6
12	1.20	180.3<br>(7.79)[2]	88.1<br>(7.42)	−71.5<br>(1.40)	67.2<br>(6.03)	150.1<br>(7.29)	44.1<br>(5.57)	34.1<br>(5.44)	32.2<br>(1.98)	20.9<br>(0.98)
$\boldsymbol{K_a}$ – isodesmic association		1.61	1.74	1.34	2.28	1.59	1.64	1.76	1.70	1.76
$\Delta\delta_{max}$ [3]		**367.0**	**174.6**	**−153.4**	**121.9**	**305.7**	89.4	67.9	64.3	41.3
the average value of $\boldsymbol{K_a}$						**1.71 ± 0.24**				

[1] $(\Delta\delta) = \delta_{\text{at lowest concentration}} - \delta_{\text{at given concentration}}$; positive values correspond to a low frequency shift, [Hz];
[2] in brackets the chemical shifts of proton signals are given, δ [ppm];
[3] $\Delta\delta_{max}$ – calculated maximum chemical shift changes related to the association process.

Table S5. The chemical shifts changes, $\Delta\delta^1$ [Hz], of the protons signals of **2** in D_2O buffer (25 mM NaCl/25 mM K_3PO_4 + TSPA-d_4) for different concentration, $\mathbf{c_2}$ [mM], pH 6.4; temp = 10°C and the isodesmic association constants $\boldsymbol{K_a}$ [mM^{-1}].

		Δδ										
	**c₂**	**ring A**			**ring B**		**ring C**		**ring D**	**ring E**		
		**H12**	**H11**	**H24**	**H23**	**H5**	**H28**	**H28**	**H14**	**H17a**	**H17b**	**H18**
1	0.003	(8.15)[2]	(7.60)	(5.10)	(1.26)	(6.20)	(4.81)	(4.35)	(7.57)	(5.66)	(5.46)	(2.02)
2	0.014	0	0	0	0	0	0	0	0	0	0	0
3	0.032	0	0	0	0	0	0	0	0	0	0	0
4	0.05	2.5	1.9	1.0	0	2.5	2.1	1.2	2.3	1.6	1.6	0.5
5	0.08	3.4	2.1	1.9	0	3.2	2.3	1.4	2.8	2.0	1.7	1.7
6	0.13	5.3	3.0	2.7	-0.6	5.0	2.7	2.3	4.5	2.5	2.5	2.1
7	0.19	5.9	3.0	2.5	-2.4	5.8	3.3	2.0	5.3	2.5	2.5	2.1
8	0.26	8.3	4.5	3.6	-2.8	8.1	4.2	2.7	7.3	3.3	3.2	2.7
9	0.33	10.7	5.4	4.8	-3.4	10.2	5.6	3.3	9.6	4.1	4.7	3.4
10	0.43	11.5	5.3	4.4	-6.4	11.0	5.0	2.6	10.1	3.6	3.9	2.2
11	0.58	16.2	7.8	6.9	-5.8	15.6	7.5	4.1	14.6	6.1	6.9	4.7
12	0.77	19.4 (8.12)[2]	8.9 (7.59)	7.5 (5.08)	-8.4 (1.28)	18.6 (6.17)	8.6 (4.80)	4.9 (4.34)	17.6 (7.54)	7.1 (5.65)	8.0 (5.45)	5.4 (2.02)
K_a – isodesmic association		0.40	0.91	0.68	0.20	0.42	0.82	1.03	0.35	1.53	0.41	0.98
$\Delta\delta_{max}$[3]		**98.0**	27.7	26.9	-67.3	**89.4**	27.7	14.8	**96.5**	15.7	40.9	15.1
the average value of ***K_a***		**0.70 ± 0.38**										

[1] $(\Delta\delta) = \delta_{\text{at lowest concentration}} - \delta_{\text{at given concentration}}$; positive values correspond to a low frequency shift, [Hz];
[2] in brackets the chemical shifts of proton signals are given, δ [ppm];
[3] $\Delta\delta_{max}$ – calculated maximum chemical shift changes related to the association process.

Aggregation studies of 1 and 2 in D_2O buffer

The self-association of compounds **1** and **2** was analyzed by NMR. The ^{1}H NMR spectra were carried out in a relatively narrow range of concentrations (3 μM - 1.2 mM) due to the limited solubility of the tested compounds. The chemical shifts of the protons at a very low concentration (5 μM for **1** and 3 μM for **2**) were taken as the experimental values for the monomer. The chemical shifts changes for a given proton for different concentrations were determined as the difference between the chemical shifts at the lowest concentration and a given concentration, expressed in Hz. The observed chemical shifts changes of protons signals for compounds **1** and **2** and association constant K_a calculated by use of the isodesmic model are given in Tables 4S and 5S, respectively (also in Table 1).

For both compounds, all protons, except H23 (see Tables 4S and 5S), were shifted to lower frequencies. However, the observed chemical shifts changes are much higher for **1** than for **2.** For **1** the largest effects were observed for aromatic H12 and H14 protons (above 150 Hz), a slightly smaller for H11, H5 and H23 (65 – 88 Hz) and the smallest for H17, H18 and H19 (20 – 40 Hz). For **2** the largest effects were also observed for aromatic H12 and H14 and additionally for H5 protons but they were smaller than for **1** (each about 20 Hz). For other protons of **2**, the observed chemical shifts changes were negligible.

Mode of binding of both diastereomers with DNA oligomer d(GCGATCGC)$_2$ based on ^{1}H NMR titration

Table S6. The chemical shifts changes, $\Delta\delta^1$ [Hz], of proton signals of **1** in D_2O buffer, pH 6 (25 mM NaCl/25 mM K_3PO_4) induced by interaction with octamer **3** in solutions of different concentrations of octamer **3**, c_3 [mM] and constant concentration of **1**; c_1 =0.29 mM, temp. 10 °C. The binding constants, $\boldsymbol{K_a}$ [mM^{-1}] were calculated based on chemical shifts changes.

Δδ [Hz]											
	c_3	**H12**	**H11**	**H14**	**H5**	**H17a**	**H17b**	**H28**	**H22**	**H18**	**H19**
1	0	(7.95)^2	(7.51)	(7.44)	(6.10)	(5.60)	(5.46)	(4.76)	(3.36)	(2.01)	(0.99)
2	0.02	3.9	6.6	2.6	11.2	4.3	4.3	5.0	10.1	3.6	2.4
3	0.03	4.3	8.5	3.2	16.3	5.1	6.3	7.1	13.1	4.7	3.3
4	0.06	6.2	13.5	4.7	28.3	8.7	10.9	12.1	19.3	8.2	5.5
5	0.09	10.5	21.3	8.1	43.8	14.7	17.3	18.4	24.6	13.3	9.2
6	0.12	13.2	26.8	9.8	56.5	17.5	21.6	23.0	31.3	16.2	10.7
7	0.18	15.5	30.8	11.9	66.9	21.3	25.4	26.9	37.1	19.9	12.8
8	0.25	23.4	42.1	18.3	89.2	30.7	35.1	36.9	49.3	26.8	18.1

9	0.37	35.9	-	27.4	120.5	42.2	-	50.2	67.10	36.5	25.2
10	0.56	46.2	69.2	35.4	143.5	51.2	57.2	60.4	80.7	44.1	30.1
11	0.77	54.5	78.4	41.3	-	57.1	63.9	67.7	87.9	48.8	34.4
12	0.87	59.0	82.7	44.2	-	60.0	67.6	71.1	93.2	52.9	35.6
$\boldsymbol{K_a}$		**1.37**	**5.34**	**1.54**	**7.72**	**4.76**	**5.56**	**5.89**	**7.57**	**5.38**	**4.89**
$\Delta\delta_{max}$ [3]		118.8	105.7	85.0	197.6	79.4	85.7	89.4	111.4	66.9	47.0
the average value of $\boldsymbol{K_a}$						**5.00±2.02 (5.89±1.07)**[4]					

[1] $\Delta\delta = \delta_A - \delta_{obs}$: the chemical shifts change of proton signals of **1,** δ_A is the chemical shifts in proton signals of **1** in the absence of octamer **3**, δ_{obs} is the observed average chemical shifts of the proton signals of **1** in the presence of octamer **3** at a given concentration of octamer **3**; positive values correspond to a low frequency shift, [Hz];

[2] in brackets, the chemical shifts δ, [ppm], of proton signals of **1** at c_1=0.29 mM, are given;

[3] $\Delta\delta_{max}$ – calculated maximum chemical shift changes related to the complexation process, [Hz];

[4] the larger value is obtained when omitting the calculations for H12 and H14 protons.

Table S7. The chemical shifts changes, $\Delta\delta^1$ [Hz], of proton signals of **2** in D_2O buffer, pH 6 (25 mM NaCl/25 mM K_3PO_4) induced by interaction with octamer **3** in solutions of different concentration of octamer **3**, c_3 [mM] and constant concentration of **2**; c_2 =0.29 mM, temp. 10 °C. The binding constants, K_a [mM^{-1}], were calculated based on chemical shift changes.

	$\Delta\delta$ [Hz]										
	c_3	**H12**	**H11**	**H14**	**H5**	**H17a**	**H17b**	**H28**	**H22**	**H23**	**H19**
1	0.00	(8.16)[2]	(7.62)	(7.57)	(6.19)	(5.65)	(5.46)	(4.81)	(3.37)	(1.27)	(0.99)
2	0.02	5.7	4.1	4.3	7.3	2.1	1.5	2.8	6.7	1.4	0
3	0.04	11.3	8.4	8.8	14.6	4.0	2.9	6.1	12.2	2.3	−0.7
4	0.07	17.3	12.9	13.6	22.7	6.1	4.1	9.1	15.5	3.4	−0.9
5	0.09	22.9	17.2	17.8	29.8	7.7	5.2	11.7	22.1	4.4	−1.1
6	0.13	31.1	23.4	24.3	40.3	10.4	7.0	15.8	28.7	5.5	−1.7
7	0.16	38.3	28..8	30.1	49.5	12.6	8.7	19.9	34.6	6.7	−2.1
8	0.22	48.6	37.6	38.1	62.3	16.0	10.8	25.4	44.7	7.6	−2.9
9	0.28	60.2	45.4	46.8	76.7	21.3	13.2	30.0	57.3	9.6	−3.9
10	0.35	69.1	51.8	53.5	87.2	-	15.3	34.3	63.5	10.5	−4.5
11	0.42	78.1	59.1	61.2	99.0	-	17.7	39.9	70.5	12.0	−5.0
12	0.65	-	74.9	77.4	-	-	20.5	49.0	88.1	14.5	−6.5
13	0.87	-	-	86.6	-	-	-	55.1	94.5	16.0	−7.2
14	1.13	123.5	-	96.2	153.0	-	-	62.5	110.9	18.4	−7.3
15	1.47	133.5	-	104.6	163.2	-	-	65.7	115.3	20.7	−7.0
$\boldsymbol{K_a}$		**3.11**	**3.52**	**3.09**	**3.64**	**1.66**[4]	**6.48**[4]	**3.40**	**3.93**	**3.86**	**4.88**
$\Delta\delta_{max}$ [3]		167.5	119.9	130.4	199.3	81.2	28.0	89.4	138.0	23.8	−8.85
the average value of $\boldsymbol{K_a}$						**3.76±1.19**					

[1] $\Delta\delta = \delta_A - \delta_{obs}$:the chemical shifts change of proton signals of **2;** δ_A is the chemical shifts in proton signals of **2** in the absence of octamer **3**, δ_{obs} is the observed average chemical shifts of the proton signals of **2** in the presence of octamer **3** at a given concentration of octamer **3**; positive values correspond to a low frequency shift, [Hz];

[2] in brackets the chemical shifts δ, [ppm], of proton signals of **2** at c_2=0.29 mM, are given;

[3] $\Delta\delta_{max}$ – calculated maximum chemical shift changes related to the complexation process, [Hz];

[4] the average value of K_a for two geminal protons is 4.07.

Molecular modeling

Table S8. Diastereomer **1** (*R*)C-5, PM7 clusters energies.

No.	Structure	PM7 ENERGY [kcal/mol]		% population
		Clust. Average	10% Clust. Min.	
1	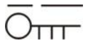	-1174.25 +/- 3.79	-1181.15	20.39
2	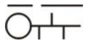	-1179.37 +/- 3.39	-1185.18	17.14
3	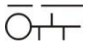	-1177.11 +/- 3.94	-1184.58	14.25
4	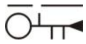	-1180.55 +/- 3.21	-1185.46	8.09
5	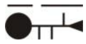	-1175.55 +/- 3.28	-1180.88	6.16
6	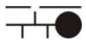	-1174.67 +/- 4.06	-1181.19	3.00
7	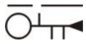	-1179.42 +/- 4.24	-1186.32	2.75
8	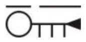	-1173.70 +/- 3.38	-1179.55	2.64
9	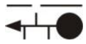	-1173.53 +/- 3.68	-1180.57	1.95
10	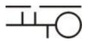	-1179.73 +/- 4.32	-1186.91	1.89
11	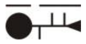	-1174.93 +/- 3.47	-1180.77	1.43
12	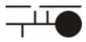	-1174.32 +/- 4.36	-1181.59	1.34
13	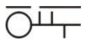	-1178.72 +/- 4.39	-1186.21	1.06
14	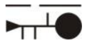	-1177.19 +/- 3.12	-1182.05	0.78
15	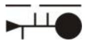	-1173.95 +/- 3.31	-1179.57	0.60
16	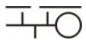	-1178.88 +/- 3.69	-1184.56	0.49
17	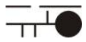	-1175.48 +/- 4.28	-1183.64	0.36
18	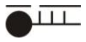	-1174.85 +/- 3.69	-1181.27	0.27

Table S9. Diastereomer **2** *(S)*C-5, PM7 clusters energies.

No.	Structure	PM7 ENERGY [kcal/mol]		% population
		Clust. Average	10% Clust. Min.	
1	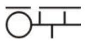	-1178.76 +/- 4.77	-1186.83	20.19
2	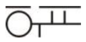	-1177.52 +/- 4.07	-1183.82	16.28
3	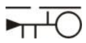	-1180.17 +/- 3.83	-1186.42	10.07
4	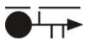	-1178.25 +/- 4.45	-1185.07	6.62
5	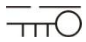	-1176.39 +/- 4.61	-1185.39	5.04
6	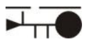	-1173.69 +/- 3.62	-1179.78	4.77

7	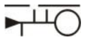	-1172.13 +/- 4.32	-1179.12	4.69
8	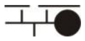	-1177.87 +/- 4.59	-1185.60	4.19
9	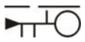	-1176.45 +/- 3.96	-1182.93	2.83
10	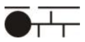	-1174.88 +/- 3.51	-1180.50	2.48
11	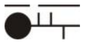	-1175.34 +/- 3.77	-1181.67	2.39
12	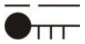	-1173.26 +/- 3.45	-1179.57	2.21
13	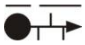	-1173.56 +/- 4.87	-1183.11	1.81
14	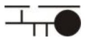	-1174.16 +/- 4.09	-1181.18	1.76
15	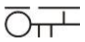	-1175.88 +/- 4.04	-1182.93	1.69
16	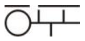	-1175.50 +/- 3.74	-1181.37	1.21
17	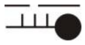	-1173.70 +/- 4.59	-1181.99	0.72
18	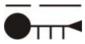	-1172.38 +/- 3.16	-1177.99	0.63

PM7 – Energies are from semi-empirical calculations. 10% Clust. Min. - are the average energies for the best 10% of conformers in current cluster.

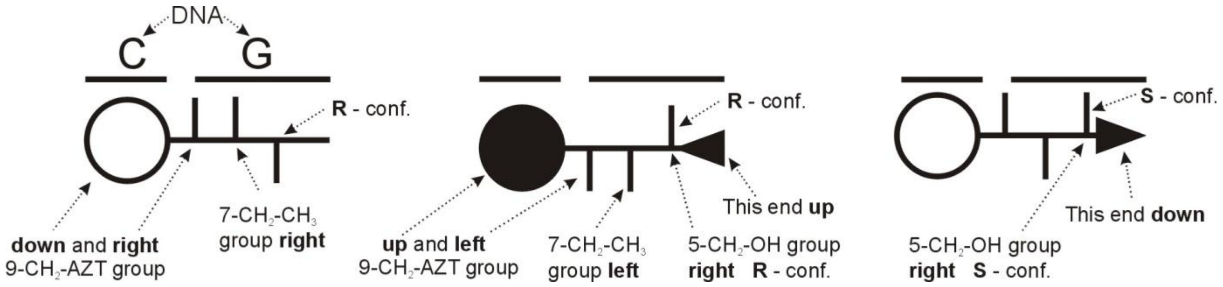

Table S10. Diastereomer **1** (*R*)C-5, PBSA and GBSA clusters energies.

No.	Structure	ENERGY [kcal/mol]		% population
		PBSA	GBSA	
1	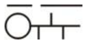	-18.41 +/- 2.10	-16.90 +/- 1.92	22.47
2	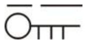	-18.56 +/- 2.08	-17.27 +/- 1.84	21.19
3	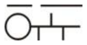	-17.78 +/- 2.25	-16.62 +/- 2.03	9.94
4	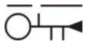	-17.97 +/- 2.18	-17.37 +/- 1.90	6.25
5	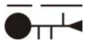	-14.91 +/- 2.42	-13.02 +/- 2.08	4.17
6	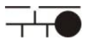	-16.04 +/- 2.14	-15.33 +/- 1.73	3.13
7	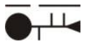	-13.54 +/- 3.34	-11.91 +/- 2.90	2.09
8	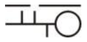	-15.57 +/- 3.80	-14.81 +/- 3.36	1.38
9	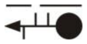	-16.10 +/- 2.22	-15.35 +/- 1.73	1.22
10	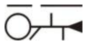	-15.79 +/- 2.37	-15.32 +/- 2.25	1.21

11	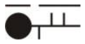	-16.86 +/- 3.19	-15.69 +/- 2.90	1.21
12	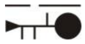	-18.56 +/- 2.93	-17.42 +/- 2.61	1.01
13	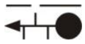	-14.97 +/- 2.66	-14.04 +/- 2.00	1.01
14	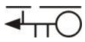	-14.57 +/- 3.74	-13.55 +/- 3.57	0.91
15	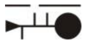	-15.30 +/- 2.80	-12.76 +/- 2.07	0.63
16	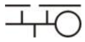	-17.02 +/- 3.05	-14.99 +/- 2.56	0.61
17	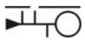	-15.25 +/- 3.04	-13.07 +/- 2.57	0.33

Table S11. Diastereomer **2** (*S*)C-5, PBSA and GBSA clusters energies.

No.	Structure	ENERGY [kcal/mol]		% population
		PBSA	GBSA	
1	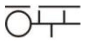	-17.05 +/- 2.66	-16.10 +/- 2.21	18.54
2	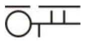	-17.55 +/- 2.73	-16.51 +/- 2.35	15.97
3	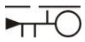	-19.96 +/- 2.18	-18.60 +/- 1.92	10.80
4	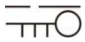	-18.03 +/- 2.16	-16.13 +/- 1.89	5.89
5	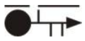	-18.34 +/- 2.29	-17.00 +/- 2.12	5.26
6	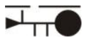	-13.69 +/- 2.51	-12.58 +/- 2.29	4.84
7	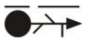	-16.96 +/- 2.29	-15.34 +/- 2.06	4.60
8	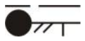	-18.79 +/- 2.89	-17.49 +/- 2.66	4.50
9	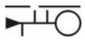	-18.08 +/- 2.36	-16.00 +/- 2.05	4.44
10	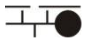	-15.16 +/- 2.63	-13.35 +/- 2.24	4.30
11	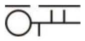	-16.61 +/- 2.28	-15.31 +/- 2.23	3.79
12	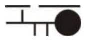	-14.59 +/- 2.84	-12.93 +/- 2.51	1.75
13	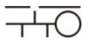	-17.36 +/- 2.08	-15.11 +/- 1.83	1.15
14	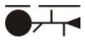	-14.26 +/- 2.07	-13.72 +/- 1.94	1.12
15	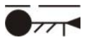	-15.74 +/- 2.80	-14.32 +/- 2.26	1.02
16	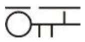	-16.98 +/- 2.14	-15.17 +/- 1.96	0.74
17	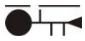	-17.27 +/- 2.62	-16.31 +/- 2.70	0.61

PBSA and GBSA energies are from Molecular Mechanics Poisson−Boltzmann and Generalized Born Surface Area Method calculations.

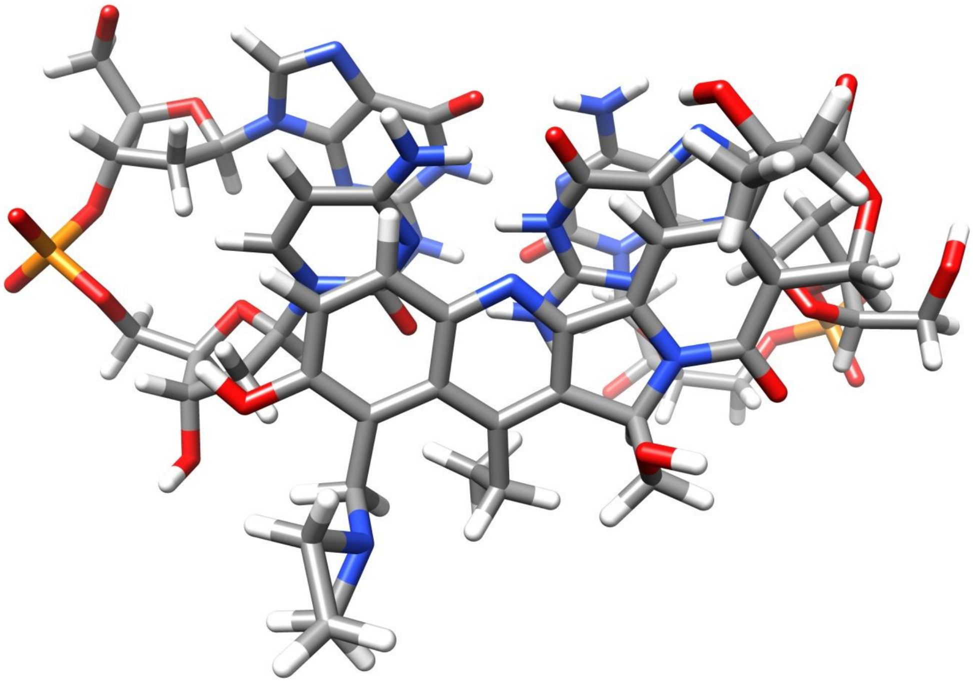

1 (5*R*, 20*S*) PBSA/GBSA Structure 1

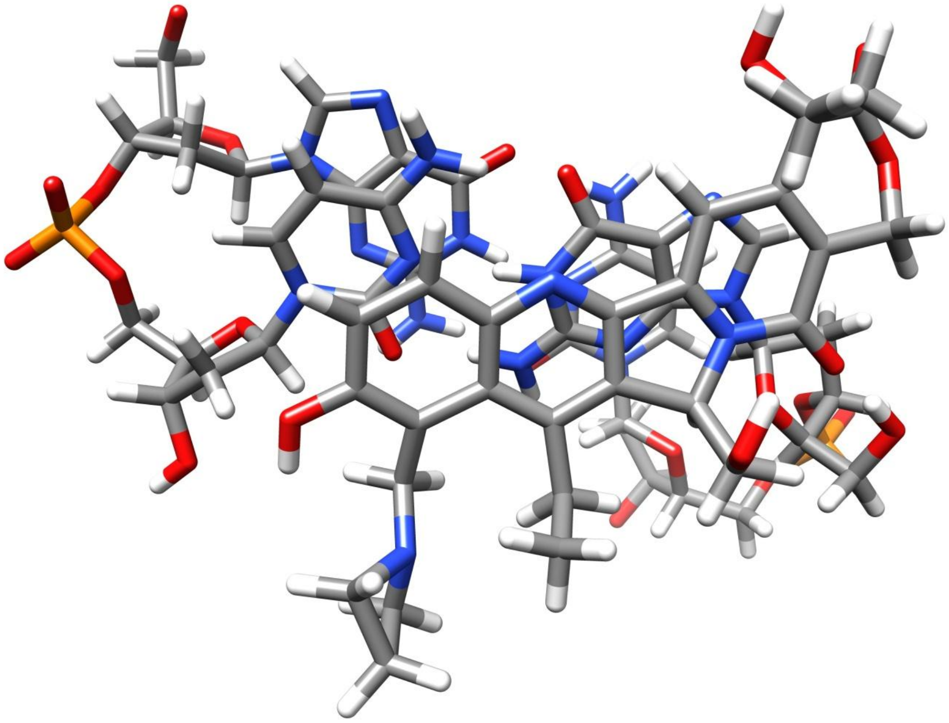

1 (5*R*, 20*S*) PBSA/GBSA Structure 2

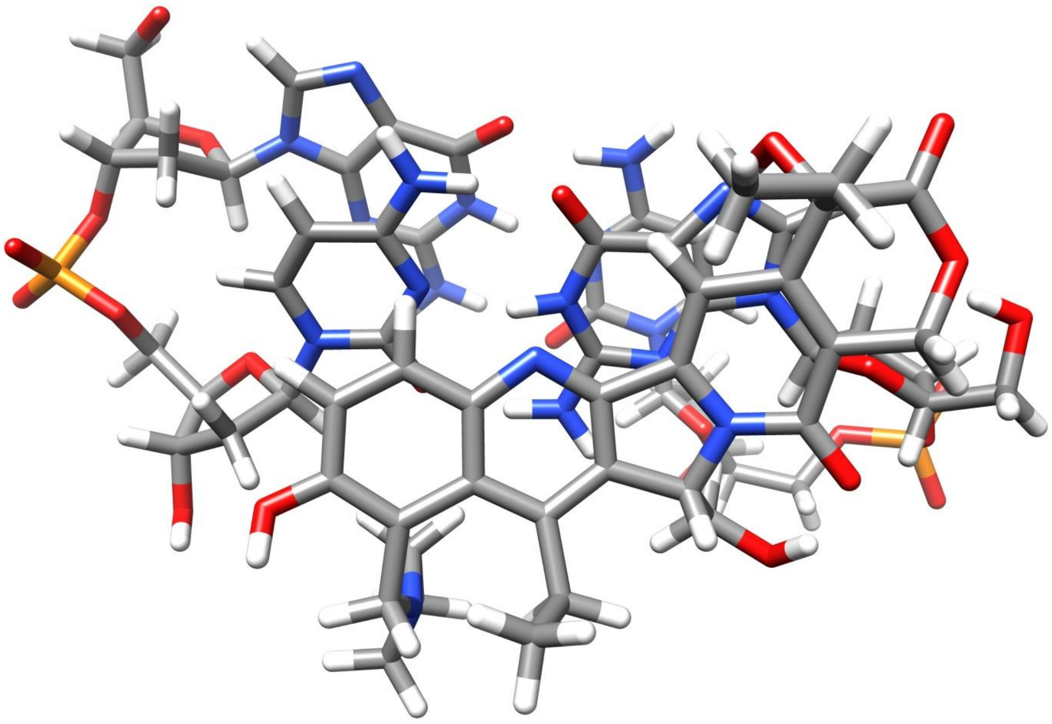

2 (5*S*, 20*S*) PBSA/GBSA Structure 1

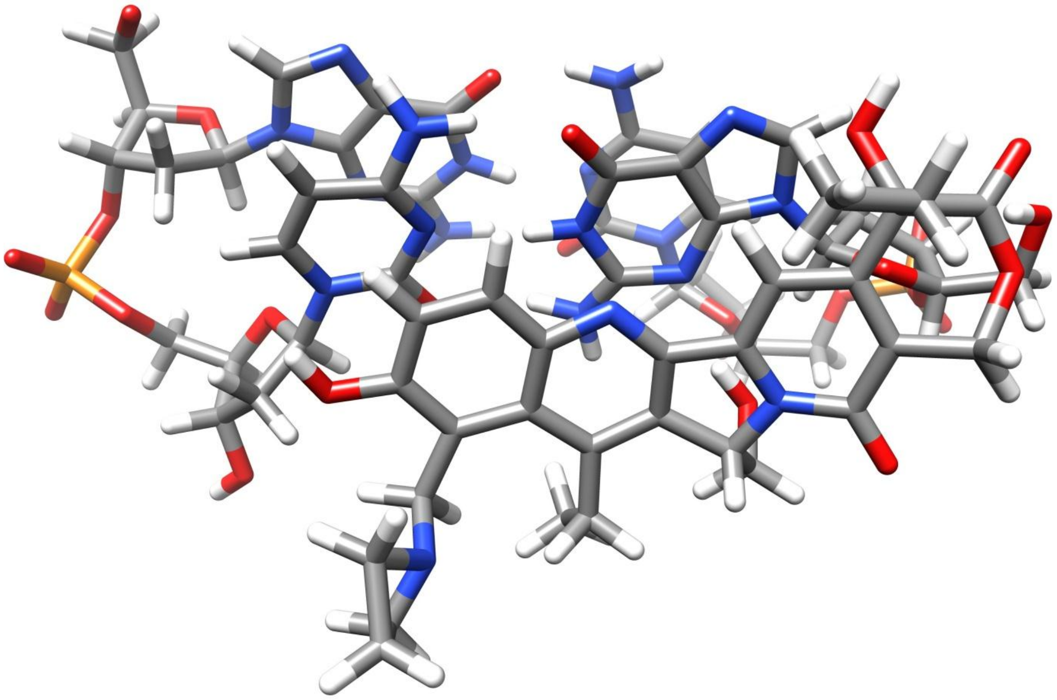

2 (5*S*, 20*S*) PBSA/GBSA Structure 2

Figure S3. Molecular model of MD of the less abundant structures of the complexes of the ultimate base pair G1-C8 of a self-complementary duplex d(GCGATCGC)$_2$ with diastereomers **1** (see Table S10, No. 1 and 2) and **2** (see Table S11, No. 1 and 2).
